# *De novo* biosynthesis of alpinetin enhanced by directed evolution of 5-O-methyltransferase

**DOI:** 10.1186/s12934-026-02996-x

**Published:** 2026-04-06

**Authors:** Bo Peng, Ziwei Wang, Lili Zhang, Matthew R. Groves, Kristina Haslinger

**Affiliations:** 1 Chemical and Pharmaceutical Biology, Groningen Research Institute of Pharmacy, University of Groningen, Antonius Deusinglaan 1, 9713AV Groningen, The Netherlands; 2 Genomics for Health in Africa (GHA), Africa-Europe Cluster of Research Excellence (CoRE), Cape Town, South Africa

**Keywords:** Alpinetin, O-methyltransferase, Directed evolution, Pinocembrin, Biosynthesis, O-methylated flavonoids, S-adenosylmethionine, *Escherichia coli*

## Abstract

**Background:**

Alpinetin ((2 S)-7-Hydroxy-5-methoxyflavan-4-one) is a natural flavonoid found in various medicinal herbs and is frequently used in Chinese patent medicines. It exhibits a wide range of bioactivities, including anti-inflammatory, cardiovascular protective, lung protective, antiviral, hepatoprotective, and antitumor effects. Alpinetin features a 5-methyl group on the A ring, a rare characteristic among methylated flavonoids. The limited abundance of alpinetin in plant biomass, its laborious extraction and purification from this biomass, and the toxicity and lack of regio- and chemoselectivity in organic synthesis render its biosynthesis in engineered microbes an attractive alternative.

**Results:**

In this study, we aimed to achieve the *de novo* biosynthesis of alpinetin in *Escherichia coli*. In a series of optimization steps, we varied the selection of pathway enzymes, plasmid configurations, medium composition, and fermentation strategies. Lastly, we applied a directed evolution campaign to the 5-O-methyltransferase to successfully enable the *de novo* biosynthesis of alpinetin in *E. coli* for the first time. Moreover, we demonstrated the ability of O-methyltransferase to methylate a broad range of flavonoid substrates, leading to the production of valuable O-methylated flavonoids.

**Conclusions:**

Our study represents the first example of alpinetin biosynthesis in a heterologous host and paves the way to produce other valuable O-methylated flavonoids enzymatically.

**Supplementary Information:**

The online version contains supplementary material available at 10.1186/s12934-026-02996-x.

## Background

**Fig. 1:**
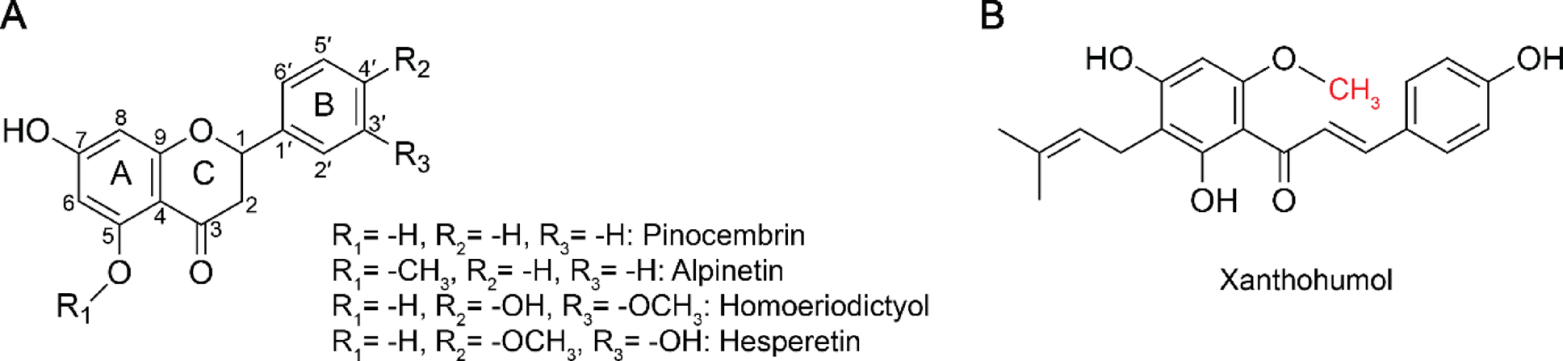
**A** Basic flavonoid structure illustrating O-methylated flavonoids mentioned in the Introduction: pinocembrin, alpinetin, homoeriodictyol, and hesperetin. **B** Xanthohumol, an open-ring chalcone with methyl and prenyl modifications, is shown separately to highlight its structural differences

 Pharmacological effects of plant-derived flavonoids have increasingly drawn attention to herbal medicines as alternative treatments for human diseases such as cancer, epilepsy, inflammation, central nervous system (CNS) disorders, diabetes, cardiovascular disorders, and to improve the immune system [1–7]. Alpinetin (7-Hydroxy-5-methoxyflavan-4-one), a rare flavonoid sourced from *Alpinia galanga* (L.), features an uncommon 5-O-methylation of the A ring (Fig. 1A). Alpinetin has been found to exhibit anti-inflammatory and antioxidant properties and has been recognized for its potential benefits in treating non-alcoholic fatty liver disease, high-fat diet-aggravated lung carcinogenesis, lipopolysaccharide/D-Galactosamine-induced liver injury, ulcerative colitis, osteoporosis, Zika virus infection, intestinal inflammation, and certain cancers [8–14].

In Traditional Chinese Medicine, alpinetin is a key component of patented drugs such as Xingqiwenzhong granule, Fufangcaodoukou tincture, Jianweizhitong tablet, and the Baikoutiaozhong pill [15]. These medications are used to treat digestive disorders, including epigastric pain, belching, nausea, vomiting, and anorexia [14, 16]. Despite the diverse bioactivities of alpinetin, its production primarily relies on extraction from plants, particularly from the seeds and rhizomes of plants from the *Zingiberaceae* family [17, 18]. However, the low extraction efficiency of the multi-step purification process limits the commercial availability of alpinetin. Moreover, the chemical synthesis of alpinetin has not yet been established, further restricting its accessibility. Recently, Sokolova et al.. reported that an O-methyltransferase (StrAOMT) from the bacterium *Streptomyces avermitilis* can methylate pinocembrin to generate alpinetin in a low yield in vitro, offering an interesting starting point for exploring microbial biosynthesis of alpinetin.[19].

O-methylation is a powerful and common tool to improve pharmacological and physicochemical properties of natural products [20, 21]. For example, homoeriodictyol and hesperetin, as shown in Fig. 1A, exhibit improved biological activities and pharmacological properties, including metabolic stability, membrane transport capability, and oral bioavailability, compared to their unmethylated counterparts [22]. Xanthohumol, illustrated in Fig. 1B, is a flavonoid with methyl and prenyl modifications and displays a range of biological activities, including cancer prevention, mitigation of diabetes, anti-inflammatory, antioxidant, and immunomodulatory effects [23, 24]. In organic chemistry, methylation reactions typically use methyl iodide, a toxic and unsustainable reagent that is harmful to the environment [25]. Furthermore, traditional chemical methylation of flavonoids often requires the use of protecting groups for the non-target hydroxyl groups to achieve selectivity, adding to the number of synthesis steps and negatively affecting the atom economy. In contrast, enzymatic methylation with S-adenosyl-L-methionine (SAM) as a methyl donor occurs under mild conditions and potentially with high regio- and chemoselectivity. In vitro, SAM analogs can even be applied to facilitate the transfer of a range of natural and non-natural alkyl groups to acceptor substrates [20, 26].

In flavonoid biosynthesis, O-methyltransferases (OMTs) can be classified into two categories: Class I OMTs, which are Mg^2+^-dependent and have a low molecular weight (23–30 kDa), and Class II OMTs, which are high molecular weight (36–43 kDa) and are metal-independent [27]. In principle, O-methylation can occur at any position; however, 4’- and 7-methylation are the most highly represented [28] as the 4’-OH group on the B ring is more reactive due to resonance stabilization from the aromatic ring. Meanwhile, the 7-OH group on the A ring is frequently methylated because it is less sterically hindered and more accessible. The 5-hydroxyl group is only rarely modified by methylation or glycosylation because it can form a hydrogen bond with the carbonyl group at carbon 3, which could alter its conformation or hinder its recognition by methyltransferases. (Fig. 1) [29].

In this study, we designed a biosynthetic pathway for *de novo* alpinetin production in *Escherichia coli* and successfully enhanced the activity of O-methyltransferase and final titer of alpinetin through directed evolution and fermentation optimization. Further studies using various flavonoids as substrates revealed that StrAOMT and its variants can O-methylate a broad range of flavonoids with specific regioselectivity for certain compounds. Our research represents the first instance of alpinetin biosynthesis in *E. coli* and will pave the way for producing valuable O-methylated flavonoids (Table 1).

## Methods

### Bacterial strains, primers, and plasmids

All bacterial strains and plasmids used in this study are listed in Table 1 and 2. Primers are given in Table 1. *E. coli* DH5alpha (New England Biolabs) was used for routine cloning and plasmid amplification, *E. coli* MG1655 K-12 (DE3) and *E. coli* BL21 (DE3) were used for protein expression and fermentation.

The genes coding for HvCHS, chalcone synthase from *Hordeum vulgare* (GenBank accession number XP_044963194); Os4CL, 4-coumarate-CoA ligase from *Oryza sativa* (GenBank accession number NP_001396278); PhCHS, chalcone synthase from *Petunia hybrida* (GenBank accession number KP284563.1); Pc4CL, 4-coumarate-CoA ligase from *Petroselinum Crispum* (GenBank accession number KX671122.1). StrAOMT, O-methyltransferase from *Streptomyces avermitilis*. MsCHI, chalcone isomerase from *Medicago sativa* (GenBank accession number P28012) are described in our previous studies [22]. The genes encoding RmXAL (GenBank accession number KR095285), aromatic amino acid ammonia lyase from *Rhodotorula mucilaginosa*; Gm4CL, 4-coumarate-CoA ligase from *Glycine max* (GenBank accession number X69955) and CsCHS, chalcone synthase from *Camellia sinensis* (GenBank accession number: D26593) were codon-optimized and synthesized (sequences provided in the Supporting Information) with the introduction of a restriction site (TWIST Bioscience, San Francisco, CA, USA).

The gene RmXAL was cloned between the BamHI and HindIII restriction sites and the gene MsCHI was cloned between the NdeI and XhoI restriction sites of pACYCDuet-1 (Novagen) yielding pACYCDuet::RmXAL-MsCHI and pACYCDuet::MsCHI, respectively. The gene for CsCHS1 was cloned between the SacI and HindIII restriction sites and the gene Gm4CL was cloned between the NdeI and XhoI restriction sites of pETDuet-1 (Novagen) yielding pETDuet::CsCHS1-Gm4CL, respectively. The gene StrAOMT was cloned between the NcoI and HindIII restriction sites of pRSFDuet-1 and pCDFDuet-1 (Novagen) yielding pRSFDuet::StrAOMT and pCDFDuet::StrAOMT, respectively. The plasmids pETDuet::PhCHS-Pc4CL, pETDuet::HvCHS-Os4CL, and pET21b::StrAOMT have been described previously [19, 22]. All plasmids were isolated with the QIAprep Spin Miniprep kit (QIAGEN) and were verified by Sanger sequencing.

### Construction of recombinant *E. coli* Strains

To create *E. coli* strains s1–s24 capable of producing flavonoids, a series of plasmid combinations were introduced into *E. coli* MG1655 (DE3) and *E. coli* BL21 (DE3) cells using electroporation. The method for preparing electrocompetent *E. coli* was adopted from Green and Sambrook [30]. After transformation, cells were cultured on selective Luria-Bertani (LB) agar. Depending on the experiment, the culture medium was supplemented with either 100 µg/L ampicillin, 20 µg/L chloramphenicol, 50 µg/L spectinomycin, 50 µg/L kanamycin, or combinations of these antibiotics to provide selective pressure for plasmid maintenance. The cells were incubated overnight at 30 °C for *E. coli* MG1655 (DE3) or at 37 °C for *E. coli* BL21 (DE3). The following day, colonies were transferred to 3 mL of selective LB liquid medium in round-bottom polystyrene tubes and cultivated at 30–37 °C and 200 rpm overnight. Subsequently, glycerol stocks (containing 50% v/v glycerol) were prepared from these cultures and stored at − 70 °C for future use.

### Multiple sequence alignment and docking study

Protein sequences were aligned with Clustal Omega (1.2.4) with default settings [31]. The alignment was visualized with ESPript [31]. Molecular docking was performed using PLANTS1.2 (https://github.com/purnawanpp/plants) with the empirical ChemPLP scoring function [32–34]. Receptor and ligand files were prepared using Open Babel version 3.1.0 and Pymol (Schrödinger, LLC).[35] The binding site was defined, and re-docking validation was conducted using the crystal structure of the *Myxococcus xanthus* OMT, SafC, in complex with S-adenosylhomocysteins (SAH), Mg^2+^, and L-dopamine (LDP) (PDB ID 5LOG) as a template [36]. Docking of pinocembrin to StrAOMT (PDB ID 8C9S) [19] was subsequently performed using the same parameterization.

### Library construction

The plasmid of pET21b::StrAOMT has been described previously [19]. Primers used for mutagenesis are listed in Supporting Table S1. Site-saturation mutagenesis was performed using QuikChange with degenerate primers of 32 codon combinations obtained from Eurofins Scientific (Luxembourg). For the PCR, 50 ng of pET21b-StrAOMTserved as the DNA template. The whole plasmid was amplified with Q5 high-fidelity DNA polymerase (New England Biolabs, Frankfurt am Main, Germany) in 50 µL reaction volume. Subsequently, 1 µL Dpn1 was added and incubated at 37 °C overnight. The resulting mixture was transformed into *E. coli* DH5α competent cells and transformants were selected on LB-agar plates, supplemented with 100 µg/mL ampicillin. Plasmids from five randomly selected colonies of each library were isolated and sequenced to ensure library quality. All.

**Table 1:** List of bacterial strains and plasmids used in this study

Strain		Relevant genotype				Reference	
*E. coli* BL21 (DE3)		F - ompT hsdSB(rB -mB -) gal dem (lIts857 indI Sam7 nin5 lavUV5-T7gene1)				Invitrogen	
*E. coli* DH5alpha		supE44 D(lacZYA-argF) U196 (F80DlacZM15) hsdR17 recA1 endA1 gyrA96 thi-1 relA1				Invitrogen	
*E. coli* K-12 MG1655 (DE3)		K-12 F^–^λ^–^ ilvG^–^ rfb-50 rph-1				37	

Identifier	Construct name		Backbone	Enzyme encoded		Source	
				MCS1	MCS2		
c1	pETDuet-1		pETDuet-1	/	/	Novagen	
c2	pACYCDuet-1		pACYCDuet-1	/	/	Novagen	
c3	pRSFDuet-1		pRSFDuet-1			Novagen	
c4	pCDFDuet-1		pCDFDuet-1	/	/	Novagen	
c5	pETDuet::PhCHS_Pc4CL		pETDuet-1	PhCHS	Pc4CL	This study	
c6	pETDuet::HvCHS_Os4CL		pETDuet-1	HvCHS	Os4CL	This study	
c7	pETDuet::CsCHS_Gm4CL		pETDuet-1	CsCHS	Gm4CL	This study	
c8	pACYCDuet::RmXAL_MsCHI		pACYCDuet-1	RmXAL	MsCHI	This study	
c9	pRSFDuet::StrAOMT		pRSFDuet-1	StrAOMT	/	This study	
c10	pCDFDuet::StrAOMT		pCDFDuet-1	StrAOMT	/	This study	
c11	pCDFDuet::StrAOMT (K212R)		pCDFDuet-1	StrAOMT (K212R)	/	This study	
c12	pCDFDuet::StrAOMT (I41L/K212R)		pCDFDuet-1	StrAOMT (I41L/K212R)	/	This study	
c13	pCDFDuet::StrAOMT (R171W/K212R)		pCDFDuet-1	StrAOMT (R171W/K212R)	/	This study	
c14	pCDFDuet::StrAOMT (I41L/R171W/K212R)		pCDFDuet-1	StrAOMT (I41L/R171W/K212R)	/	This study	
c15	pACYCDuet::MsCHI		pACYCDuet-1	/	MsCHI	This study	
c16	pET21b::StrAOMT		pET21b	StrAOMT	/	19	
c17	pET21b::StrAOMT (K212R)		pET21b	StrAOMT (K212R)	/	This study	
c18	pET21b::StrAOMT (I41L/K212R)		pET21b	StrAOMT (I41L/K212R)	/	This study	
c19	pET21b::StrAOMT (R171W, K212R)		pET21b	StrAOMT (R171W, K212R)	/	This study	
c20	pET21b::StrAOMT (I41L/R171W/K212R)		pET21b	StrAOMT (I41L/R171W/K212R)	/	This study	

^*^MCS1 and 2, multiple cloning site (each with its own T7 promoter and terminator); RmXAL, XAL from *Rhodotorula mucilaginosa*; Gm4CL, 4CL from *Glycine max*; CsCHS, CHS from *Camellia sinensis*; PhCHS, CHS from *Petunia hybrida*; MsCHI, CHI from *Medicago sativa*; Pc4CL, 4CL from *Petroselinum crispum*; HvCHS, CHS from *Hordeum vulgare*; Os4CL, 4CL from *Oryza sativa*; StrAOMT, OMT from *Streptomyces avermitilis* were codon-optimized for expression in *E. coli*

**Table 2:** List of alpinetin-producing strains used in fermentation experiments

Identifier	Construct name	enzymes expressed	E. coli strain
s1	c5, c8, c9	RmXAL, Pc4CL, PhCHS, StrAOMT, MsCHI	MG1655 (DE3)
s2	c6, c8, c9	RmXAL, Os4CL, HvCHS, StrAOMT, MsCHI	MG1655 (DE3)
s3	c7, c8, c9	RmXAL, Gm4CL, CsCHS1, StrAOMT, MsCHI	MG1655 (DE3)
s4	c5, c8, c9	RmXAL, Pc4CL, PhCHS, StrAOMT, MsCHI	BL21 (DE3)
s5	c7, c8, c9	RmXAL, Gm4CL, CsCHS1, StrAOMT, MsCHI	BL21 (DE3)
s6	c5, c8, c10	RmXAL, Pc4CL, PhCHS, StrAOMT, MsCHI	BL21 (DE3)
s7	c7, c8, c10	RmXAL, Gm4CL, CsCHS1, StrAOMT, MsCHI	BL21 (DE3)
s8	c1, c2, c10	StrAOMT	BL21 (DE3)
s9	c4, c5, c8	RmXAL, Pc4CL, PhCHS, MsCHI	BL21 (DE3)
s10	c4, c7, c8	RmXAL, Gm4CL, CsCHS1, MsCHI	BL21 (DE3)
s11	c7, c8, c11	RmXAL, Gm4CL, CsCHS1, StrAOMT (K212R), MsCHI	BL21 (DE3)
s12	c7, c8, c12	RmXAL, Gm4CL, CsCHS1, StrAOMT (I41L/K212R), MsCHI	BL21 (DE3)
s13	c7, c8, c13	RmXAL, Gm4CL, CsCHS1, StrAOMT (R171W/K212R), MsCHI	BL21 (DE3)
s14	c7, c8, c14	RmXAL, Gm4CL, CsCHS1, StrAOMT (I41L/R171W/K212R), MsCHI	BL21 (DE3)
s15	c7, c14, c15	Gm4CL, CsCHS1, StrAOMT (I41L/R171W/K212R), MsCHI	BL21 (DE3)
s16	c16	StrAOMT	BL21 (DE3)
s17	c17	StrAOMT (K212R)	BL21 (DE3)
s18	c18	StrAOMT (I41L/K212R)	BL21 (DE3)
s19	c19	StrAOMT (R171W, K212R)	BL21 (DE3)
s20	c20	StrAOMT (I41L/R171W/K212R)	BL21 (DE3)
s21	c3, c5, c8	RmXAL, Pc4CL, PhCHS, MsCHI	BL21 (DE3)
s22	c4, c5, c8	RmXAL, Pc4CL, PhCHS, MsCHI	BL21 (DE3)
s23	c3, c7, c8	RmXAL, Gm4CL, CsCHS, MsCHI	BL21 (DE3)
s24	c4, c7, c8	RmXAL, Gm4CL, CsCHS, MsCHI	BL21 (DE3)

other colonies were resuspended together in LB medium and plasmid DNA was isolated and used to generate the mutant library in *E. coli* BL21 (DE3).

### Screening of StrAOMT variants using cell-free extract

Screening of StrAOMT variants was performed with cell-free extract (CFE) in a 96-deep well format. The library plasmid DNA was transformed into electroporation competent *E. coli* BL21(DE3) and grown on LB agar plates supplemented with 100 µg/mL ampicillin at 37 °C overnight. Bacterial colonies were picked from these agar plates with sterile toothpicks and used to inoculate 125 µL LB medium with 100 µg/mL ampicillin in a 96-well plate. As control, three wells were inoculated with *E. coli* BL21 (DE3) cells harboring pET21b::StrAOMT, pET21b::StrAOMT (K212R), pET21b::StrAOMT (I41L/K212R), or pET21b::StrAOMT (R171W/K212R). The plates were sealed with sterile gas-permeable seals (Breathe-Easy, Diversified Biotech, Boston, MA, USA) and starter cultures were grown at 37 °C, 220 rpm overnight. From this overnight culture, 25 µL were used to inoculate 1.2 mL TB medium supplemented with 100 µg/mL ampicillin in 96-deep well plates that were placed at 37 °C, 220 rpm until an optical density ((λ = 600 nm); OD_600_) of 0.4–0.6. Isopropyl-β-D-1-thiogalactopyranoside (IPTG, final concentration, 1 mM) was subsequently added for induction of protein expression at 30 °C, 220 rpm overnight. The remainder of each overnight culture was used to prepare glycerol stocks stored at − 70 °C for later use. After overnight incubation, the cells in 96-deep well plates were collected by centrifugation (30 min, 3,100 x g), the supernatant was removed and the cell pellets were frozen for 45 min at − 70 °C. The cells were then thawed and incubated for 45 min at 30 °C in 200 µL lysis buffer containing 25 mM Tris-HCl pH 7.5, 1 mg/mL lysozyme and 5 µg/mL Benzonase (Novagen). The insoluble cell fraction was removed by centrifugation (45 min, 3,100 x g) and 90 µL of the CFE was transferred into a 96-deep well plate for activity screening of StrAOMT variants. Enzymatic reactions were performed with 90 µL CFE and 10 µL of a reaction mix including 1 mM pinocembrin, 4 mM S-adenosylmethionine (SAM), 20 mM MgCl_2_ for 6 h at 37 °C. The reactions were quenched with 100 µL methanol and insoluble proteins were removed by centrifugation (45 min, 3,100 x g). The supernatants were transferred into a new 96-well plate for reverse-phase HPLC analysis. For each library, the three wells exhibiting the highest activity were selected for plasmid DNA isolation and sequencing.

### *E. coli* whole-cell biotransformation experiments

For 48 well plate fermentations, the alpinetin-producing strains were streaked in triplicate from glycerol stocks onto selective LB agar plates. After overnight incubation at 30 °C, single colonies were inoculated into starter cultures of 3 mL of selective LB medium in round-bottom polystyrene tubes and grown with shaking at 200 rpm at 30 °C overnight. The next day, the cultures were diluted 1:100 into 3 mL seed cultures of modified selective 4-morpholinepropanesulfonic acid (MOPS) medium for 24 h at 30 °C. These were used to inoculate working cultures of 1 mL at an OD_600_ of 0.05 in a 48 flower-shaped well plate (m2p-labs, Germany) and incubated at 30 °C, 900 rpm in an Eppendorf Thermomixer. Isopropyl-β-d-1-thiogalactopyranoside (IPTG) (final concentration, 1 mM), methionine (final concentration, 0–10 mM), phenylalanine (final concentration, 0.1–3.1 mM), and cerulenin (final concentration, 20 µg/mL) were added to the cell culture after 0–6 h, and incubation was continued for 36 h. Modified mops medium composition (1x) prepared from sterile stocks: MOPS medium, Trace Mineral Supplement (ATCC MD-TMS, used as 200 x stock), vitamin mix (from 100x stock; final: riboflavin 0.84 mg/L, folic acid 0.084 mg/L, nicotinic acid 12.2 mg/ L, pyridoxine 2.8 mg/L, and pantothenic acid 10.8 mg/L), biotin (from 1000 x stock; final: 0.24 mg/L), thiamine (from 1470 x stock; final: 340 mg/L), 4% (w/v) glucose (from 50% (w/v) stock), MgCl_2_ (from 500x sterile stock in water, final 2 mM).

For fermentation in shake flasks, three single colonies of each respective strain were inoculated into selective LB medium and incubated overnight at 37 °C. The next day, 1 mL of the starter culture was inoculated into 50 mL of fresh, selective TB medium. IPTG (final concentration, 1 mM) was added to the cultures when the OD_600_ reached 0.4 − 0.6 for protein expression and cultures were subsequently incubated at 30 °C for 16 h. The cells were collected by centrifugation for 30 min at 3,100 x g (Eppendorf 5920R, Germany) and 4 °C. Two cell pellets were combined and resuspended in 5 mL of fresh modified MOPS medium, which included 1 mM phenylalanine, 0.1 mM methionine, 1 mM IPTG, 4% (w/v) glucose (from 50% (w/v) stock), and 2 mM MgCl_2_ for a further 36 h of fermentation at 30 °C. Samples were taken for quantification of metabolites after 36 h of fermentation. To determine the concentrations of cinnamic acid, pinocembrin, and alpinetin, 1 mL of whole fermentation culture was taken from either the 48-well plate or the flask and mixed with 1 mL of methanol solution (100% methanol containing 0.1% (v/v) TFA). The methanol‑treated sample was then vortexed and centrifuged at 10,000 × g for 10 min, and the resulting supernatant was used for HPLC analysis. To analyze the alpinetin content in the fermentation culture, 1 mL samples from the 48-well plate or 2 mL samples from the flask were each extracted twice with 2 mL or 4 mL of ethyl acetate, respectively. The organic phase was collected and evaporated. After evaporation, 1 mL of 50% (v/v) methanol (in water) was added to dissolve the crude extract, which was then filtered using a 0.45 μm PTFE syringe filter to remove insoluble particles before LC-MS analysis. Samples were stored at − 20 °C until analysis.

### Analysis of target compounds

The authentic standards for pinocembrin, alpinetin, and S-adenosylmethionine (SAM) were purchased from BLDpharm (Shanghai, China). Fatty acid synthase (FAS) inhibitor cerulenin was purchased from Enzo Life Sciences (USA). Cinnamic acid, caffeic acid, phenylalanine, thiamine, kaempferol, luteolin, quercetin, chrysin, naringenin, hesperetin, eriodictyol, homoeriodictyol, phloretin, prunin, naringenin 5-methyl ether, and biotin were purchased from Sigma-Aldrich (USA). Amentoflavone, myricetin, eupatorin were purchased from Extrasynthese (France).

The choice of analytical method was based on metabolite abundance, sample complexity, and chemical characteristics: HPLC was used for high-abundance or simple mixtures, whereas LC–MS and high-resolution MS/MS were employed for complex samples and for unambiguous structural confirmation. Quantification of cinnamic acid, pinocembrin, and alpinetin from the enzymatic reactions and fermentation cultures was achieved by high-performance liquid chromatography (HPLC), with a Shimadzu LC-10AT system equipped with a SPD-20 A photodiode array detector (PDA). The samples were analyzed using 20 µL injections and separation over an Agilent Eclipse XDB-C18 (5 μm, 4.6 × 150 mm) column. Samples from enzymatic reactions were separated by isocratic elution with 45% mobile phases A (water + 0.1% (v/v) trifluoroacetic acid (TFA)) and 55% B (acetonitrile + 0.1% (v/v) TFA) at a flow rate of 1 mL/min. To separate metabolites from 48 well plate and flask fermentation, the following gradient was used: 15% B for 3 min, 15–90% B over 6 min; 90% B for 2 min; 90 − 15% B over 3 min, 15% B for 4 min; flow rate: 1 mL/min. Cinnamic acid, pinocembrin, and alpinetin were identified by comparison to authentic standards. The peak areas were integrated and converted to concentrations based on calibration curves with the authentic standards (Figure S1).

The identity of alpinetin and other O-methylated flavonoid derivatives were assessed by liquid chromatography−mass spectrometry (LC-MS) with a Waters Acquity Arc HPLC-MS system equipped with a 2998 PDA detector and a QDa single-quadrupole mass detector. Samples (2 µL) were injected into and separated over an Xbridge BEH C18 (3.5 μm, 2.1 × 50 mm) column with the following mobile phases: A: water + 0.1% (v/v) formic acid (FA); B, acetonitrile + 0.1% (v/v) FA. The following gradient was used: 5% B for 2 min, 5 − 90% B over 3 min, 90% B for 2 min, 90 − 5% B over 3 min; flow rate: 0.5 mL/min. MS analysis was carried out in positive ionization mode, with the following parameters: probe temperature of 600 °C; capillary voltage of 1.0 kV; cone voltage of 15 V; scan range 100–1250 m/z.

The identity of alpinetin and other O-methylated flavonoid derivatives was further assessed by High-resolution tandem MS analyses, which were performed with a Shimadzu Nexera X2 HPLC system with binary LC20ADXR interfaced to a QExactive Plus Hybrid Quadrupole-Orbitrap Mass Spectrometer (Thermo Scientific). The column, injection volume, flow rate, mobile phases, and LC method were used as described above for HPLC-MS. MS and MS/MS analyses were performed with electrospray ionization in positive mode at a spray voltage of 3.5 kV, a sheath gas pressure of 60 psi, and an auxiliary gas flow of 11 arbitrary units. The ion transfer tube temperature was 300 °C. Spectra were acquired in data-dependent mode with a survey scan at m/z 100 − 1650 at a resolution of 70,000 followed by MS/MS fragmentation of the top 5 precursor ions at a resolution of 17,500. A normalized collision energy (NCE) of 35 or a mix of 30, 40, and 55 were used for fragmentation, and fragmented precursor ions were dynamically excluded for 10 s.

### Protein expression, purification, and activity tests

#### Expression

*E. coli* BL21(DE3) harboring the plasmids pET21b::StrAOMT, pET21b::StrAOMT (K212R), pET21b::StrAOMT (I41L/K212R), pET21b::StrAOMT (R171W/K212R), pET21b::StrAOMT (I41L/R171W/K212R) were inoculated in 3 mL LB (containing 100 µg/mL ampicillin) for overnight cultivation at 37 °C and 200 rpm. The next day, 1 mL of overnight cell culture was used to inoculate 1 L of selective TB medium at 37 °C and 200 rpm in a 5 L glass Erlenmeyer flask. When the OD_600_ reached 0.4–0.6, 1 M IPTG (final concentration, 1mM) was added for induction of protein expression. The TB medium composition (1 L) consisted of 20 g tryptone, 24 g yeast extract, 4 g glycerol, 2.3 g potassium dihydrogen phosphate, 12.5 g dipotassium phosphate, and 100 µg/mL ampicillin.

#### Purification

All steps were performed with cooled buffers at 4 °C. Cells were harvested by centrifugation at 3,100 x g for 20 min. The cell pellet was resuspended in 20 mL of lysis buffer (50 mM Tris − HCl and 200 mM NaCl, pH 7.5). The cells were disrupted by sonication for 4 × 40 s (with a 5 min rest interval between each cycle) at 60 W output. The unbroken cells and debris were removed by centrifugation (10,000 x g for 1 h). The supernatant was filtered through a syringe filter (pore diameter, 0.45 μm) and incubated with 3 mL of Ni^2+^-NTA sepharose resin, which had previously been equilibrated with lysis buffer, in a small column at 4 °C for 18 h with agitation. The unbound proteins were eluted from the column using gravity flow. The column was first washed with lysis buffer (15 mL) and then with buffer A (30 mL, 50 mM Tris − HCl, 200 mM NaCl, and 30 mM imidazole, pH 7.5). Retained proteins were eluted with buffer B (5 mL, 50 mM Tris − HCl, 300 mM NaCl, and 500 mM imidazole, pH 7.5). Fractions were analyzed by separation using sodium dodecyl sulfate polyacrylamide gel electrophoresis (SDS-PAGE)(4 − 12% polyacrylamide) and staining with the InstantBlue Coomassie Protein stain (Abcam, UK). Fractions containing O-methyltransferase were pooled and loaded onto a HiLoad 16/600 Superdex 200 pg column, which had previously been equilibrated with buffer C (180 mL, 10 mM HEPES, 200 mM NaCl buffer, 5% glycerol, and 10 mM DTT, pH 7). Elution was performed in buffer C at 1 mL/min for 1.2 column volumes. Fractions were collected and analyzed by SDS-PAGE. The purified enzymes were concentrated with centrifugal devices with Omega membrane 30 K (Pall, USA) and stored at − 70 °C until further use.

#### Activity tests

The in vitro OMT reactions were conducted in a buffer containing 25 mM Tris-HCl (pH 7.5), 2 mM MgCl₂, 1 mM SAM, 0.5 mM substrate, and 5 µM enzyme in a total reaction volume of 100 µL. Reactions were incubated at 37 °C for 6 h. For kinetic characterization of StrAOMT wild-type and the StrAOMT I41L/R171W/K212R variant, reactions were performed under similar conditions with substrate concentrations ranging from 6.25 to 625 µM and 50 µM enzyme in a total volume of 100 µL. Incubations were carried out at 37 °C for 20, 40, and 60 min. Substrate scope screening was conducted in a buffer containing 25 mM Tris-HCl (pH 7.5), 2 mM MgCl₂, 1 mM SAM, 500 µM substrate, and 50 µM enzyme in a final volume of 100 µL, with incubation at 37 °C for 12 h. Reactions were initiated by the addition of enzyme (or water for the “no OMT” control), incubated at 37 °C, then quenched with 100 µL 100% methanol containing 0.1% (v/v) TFA. Samples were centrifuged and stored at −20 °C until analysis.

## Results

### Biosynthesis of alpinetin

**Fig. 2:**
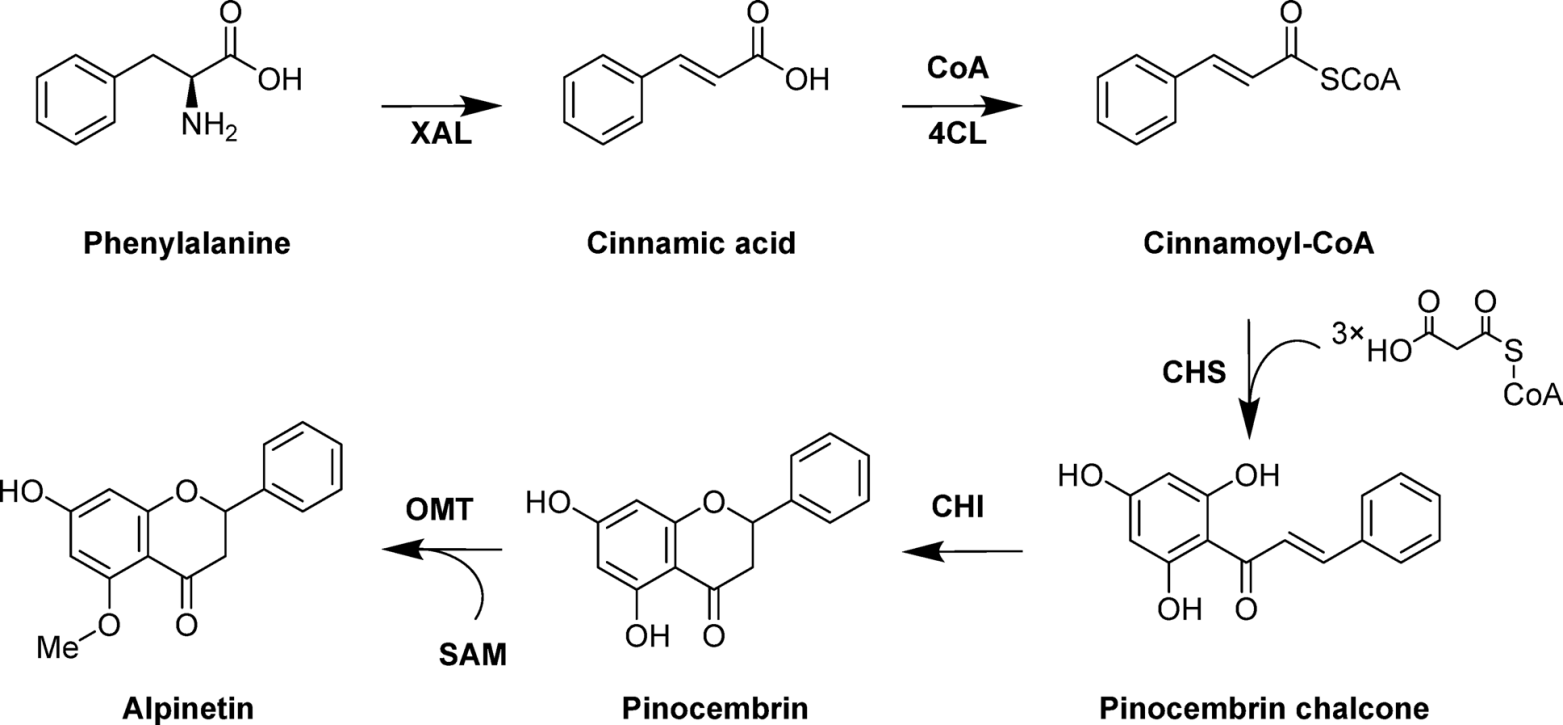
The proposed biosynthesis pathway for alpinetin. *XAL* aromatic amino acid ammonia lyase, *4CL* 4-coumarate, *CoA* ligase, *CHS* chalcone synthase, *CHI* chalcone isomerase, *OMT* O-methyltransferase

Several studies have documented the production of pinocembrin, the precursor of alpinetin, in *E. coli* and *Saccharomyces cerevisiae *[38–42]. It can be obtained from phenylalanine via several enzymatic steps catalyzed by aromatic amino acid ammonia lyase (XAL), 4-courmate: CoA ligase (4CL), chalcone synthase (CHS), and chalcone isomerase (CHI), respectively. Based on the results of previous studies on cinnamic acid production in *E. coli*, ^43^ we chose the aromatic amino acid ammonia lyase from *Rhodotorula mucilaginosa* (RmXAL) for cinnamic acid production. To produce pinocembrin chalcone, we tested different combinations of CHS and 4CL enzymes, namely PhCHS and Pc4CL, known for their high efficiency of naringenin production [44]; HvCHS and Os4CL, previously utilized for synthesis of methylated flavonoids [22]; and CsCHS1 and Gm4CL, previously recognized for their capacity to generate significant amounts of pinocembrin [40]. Lastly, we used chalcone isomerase from *Medicago sativa* for cyclization of pinocembrin chalcone and the recently characterized O-methyltransferase from *Streptomyces avermitilis*, StrAOMT (Figure. 2) [19].

**Fig. 3:**
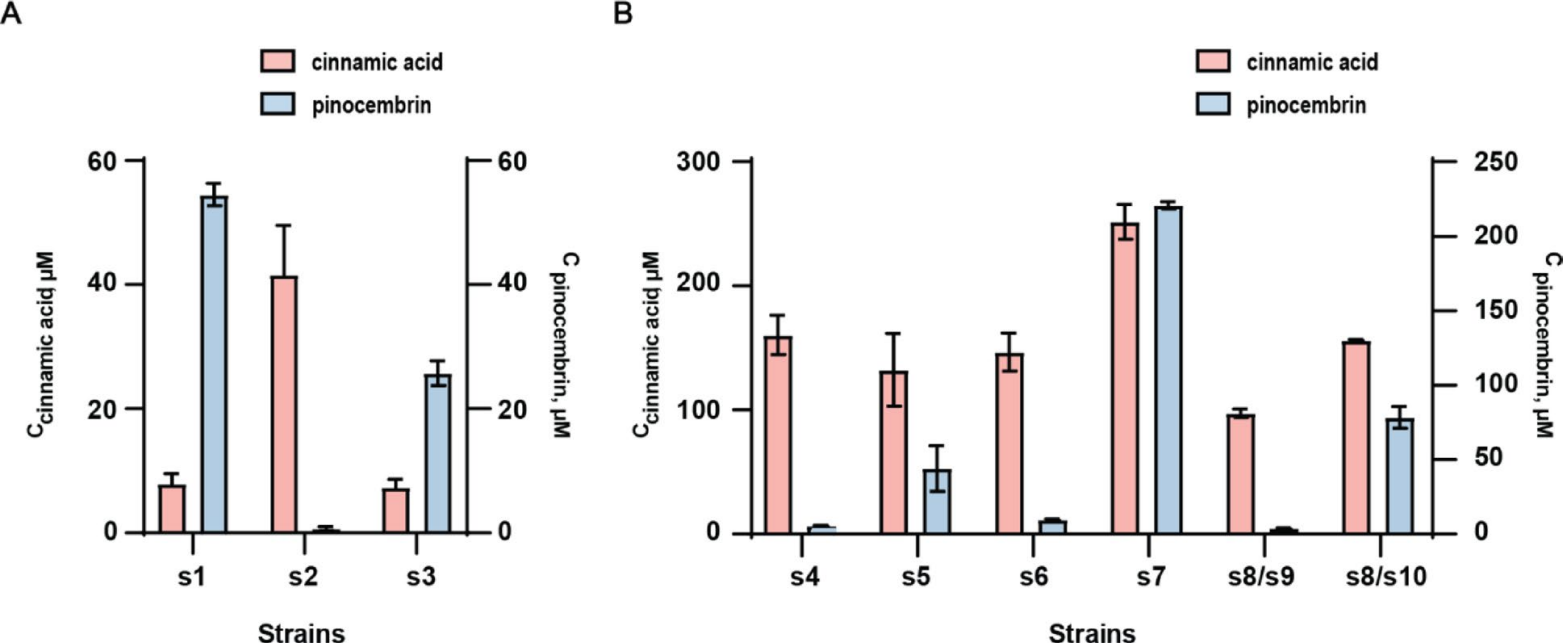
**A** Fermentation of *E. coli* MG1655 (DE3) harboring the recombinant flavonoid biosynthesis pathways. Titers of cinnamic acid (red, left axis) and pinocembrin (blue, right axis) were determined 48 h after induction of enzyme expression. **B** Biotransformation in resting *E. coli* BL21 (DE3) harboring the recombinant flavonoid biosynthesis pathway. s7 was seen to produce trace amounts of alpinetin, as detected by LC-MS. Bars represent mean +/- SD, *n* = 3

Strains s1-s3 were constructed by co-transformation of the resulting plasmids into *E. coli* MG1655 (DE3) (see Table 2). Initial fermentations were carried out in 1 mL modified MOPS minimal medium, supplemented with 3 mM phenylalanine. The results indicated that the combination of PhCHS and Pc4CL (s1) produced the highest titer of pinocembrin (approximately 56 µM (Fig. 3A)). In contrast, combining HvCHS and Os4CL (s2) resulted in a high titer of cinnamic acid but low titer of pinocembrin (0.5 µM). Additionally, the combination of CsCHS1 and Gm4CL (s3) produced around 27 µM of pinocembrin, but no alpinetin was observed in any of the fermentations. Since s2 produced less pinocembrin compared to the other strains, the combination of HvCHS and Os4CL was excluded from further experiments.

Subsequently, we conducted a series of experiments for s1 to optimize (co-)substrate (methionine, phenylalanine) feeding time and concentration to enhance pinocembrin production and facilitate the generation of alpinetin. Our findings revealed that a lower concentration of phenylalanine (0.1 mM), the absence of methionine, and a substrate feeding time of 3 h enhanced the titer of pinocembrin, achieving a highest titer of approximately 92.6 µM (Figure S2). However, alpinetin was still not detected in the final fermentation culture.

Initially, we attributed the lack of alpinetin production to low availability of endogenous SAM in vivo, or low concentration of the OMT enzyme. Fermentations were initially performed in MOPS minimal media, which imposes a metabolic burden on *E. coli* and may lead to lower protein expression levels and lower ATP generation. ATP is crucial for SAM biosynthesis as it provides the adenosyl group and serves as an energy donor [45]. To overcome this limitation, we implemented a two-step protocol to decouple cell growth and protein expression from the flavonoid production phase. Specifically, cells were first cultivated in rich media to achieve high protein expression, then harvested and resuspended in modified MOPS medium as resting cells for biotransformation. This approach minimizes cell growth and redirects metabolic resources, including ATP and SAM, toward flavonoid production rather than biomass formation. We co-transformed the plasmids into *E. coli* BL21 (DE3) to generate s4 and s5 and subjected them to the two-step protocol. Although we still did not detect alpinetin in the culture, our experiments revealed that the combination of CsCHS and Gm4CL (s5, 46.5 µM) produced more pinocembrin than the combination of PhCHS and Pc4CL (s4, 6.5 µM) in this strain under these experimental conditions (Fig. 3B).

In our initial plasmid configuration StrAOMT was expressed from the high copy-number plasmid pRSFDuet-1, which imposes a metabolic burden as demonstrated in Figure S3 and reported previously [46]. Because strains carrying the moderate–copy number plasmid pCDFDuet-1 showed improved cinnamic acid and pinocembrin production when co-expressed with either the PhCHS/Pc4CL or CsCHS/Gm4CL enzyme combinations, we switched StrAOMT expression to this lower–copy vector to generate strains s6 and s7. This modification increased pinocembrin titers to 14.75 µM (s6) and 205 µM (s7) (Fig. 3B). In s7, we found trace amounts of alpinetin detected by low resolution mass spectrometry (around 0.25 µM) and further confirmed by high-resolution tandem MS analyses (Figure S4).

To further alleviate the metabolic burden, we employed a two-strain strategy in which the pinocembrin biosynthetic module and the OMT module were expressed in separate strains. Specifically, strain s8 expressed only StrAOMT at the highest possible level, while strains s9 and s10 carried the genes encoding RmXAL, MsCHI, and either Pc4CL/PhCHS or Gm4CL/CsCHI, respectively. After 16 h of induction in TB medium, the strains were harvested, and their cell pellets were combined and resuspended in selective MOPS medium supplemented with 1 mM phenylalanine, 0.1 mM methionine, 1 mM IPTG, 4% (w/v) glucose, and 2 mM MgCl_2_ for biotransformation. Despite these efforts, the final titers of pinocembrin were lower than in s6 and s7, and no alpinetin was detected (Fig. 3B).

To exclude the possibility that OMT was entirely dysfunctional in our engineered strains, we then tested the ability of s7 and s8 to methylate caffeic acid, a previously reported substrate of StrAOMT with the same two-step biotransformation protocol.[19, 21] We used 1 mM caffeic acid as a substrate and obtained 0.12 mM ferulic acid and 0.036 mM isoferulic acid in the fermentation culture of s7, and 0.22 mM ferulic acid and 0.04 mM isoferulic acid of s8 (Figure S5). The molar yield of products from s8 is comparable to results obtained in our previous study [21]. This indicates that StrAOMT is expressed and active in these strains with sufficient SAM supply. Thus, we conclude that StrAOMT is likely the catalyst generating the trace amounts of alpinetin in the fermentation of s7, however, its low affinity and/or catalytic efficiency for this substrate limits product titers.

### Site-saturation mutagenesis of 5-O-methyltransferase

After conducting a series of optimizations in the choice of pathway enzymes, plasmid configurations, medium composition, and fermentation strategies, alpinetin production was still very low. We attribute this issue to the low activity of the O-methyltransferase. Previous studies on class I O-methyltransferases have indicated that they carry a conserved catalytic triad of lysine- asparagine-aspartate (K-N-D) and that the adjacent hydroxyl groups of the substrate coordinates the metal ion during catalysis [19, 47, 48].

**Fig. 4:**
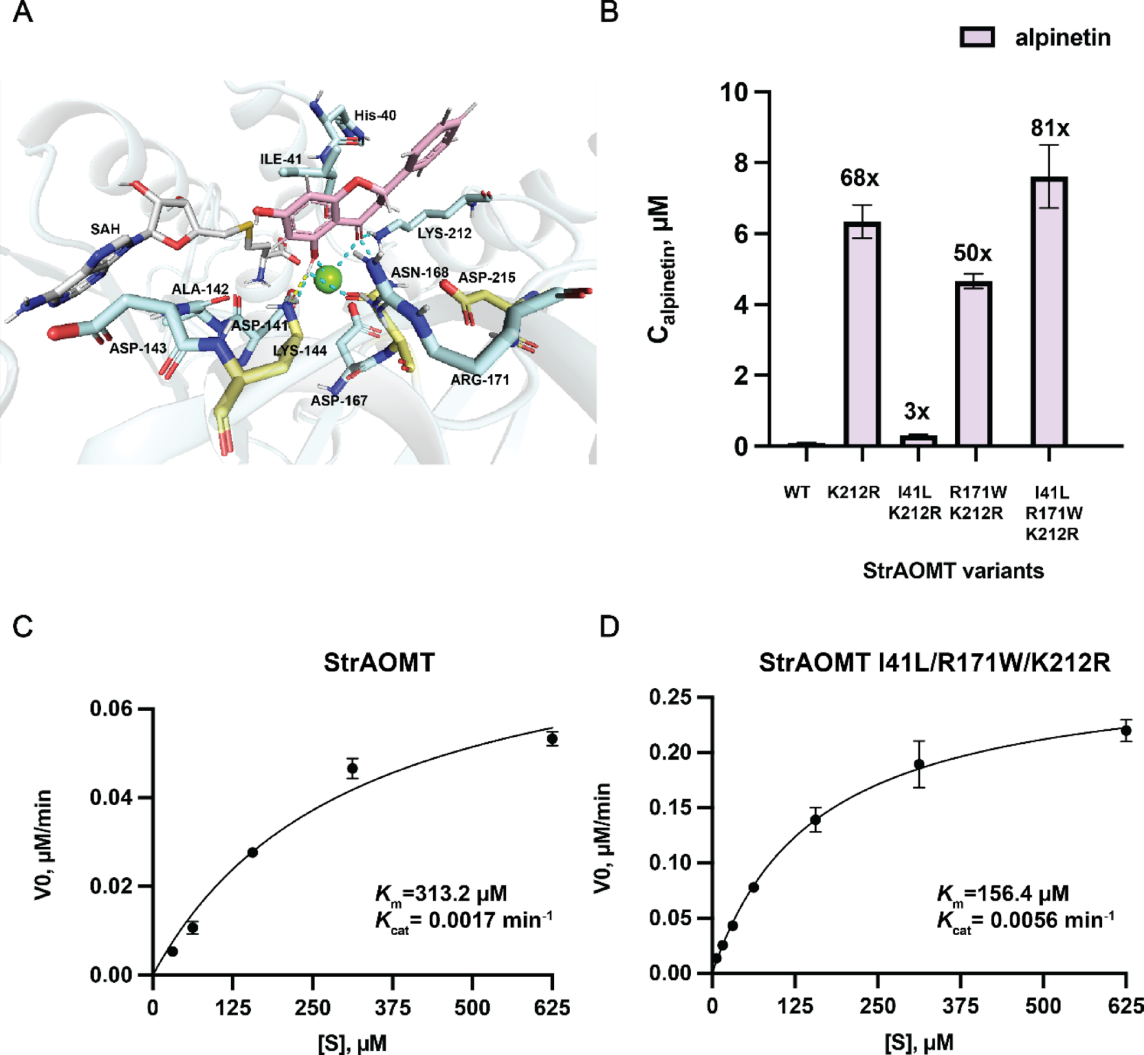
**A** Docking model of StrAOMT (PDB ID 8C9S) with pinocembrin (active site residues surrounding the bound SAH and the docked pinocembrin shown as stick representation with atom coloring: blue - nitrogen, red – oxygen, yellow – sulfur, grey - SAH carbon, light blue – StrAOMT carbon, pink – pinocembrin carbon). **B** Concentration of alpinetin produced in in vitro reactions with purified StrAOMT variants (reaction conditions: 5 µM enzyme, 500 µM pinocembrin, 2 mM SAM, 2 mM Mg^2+^ in Tri-HCL buffer (pH 7.5) at 37 °C for 6 h). **C** and **D** Kinetic characterization of StrAOMT wild type and StrAOMT I41L/R171W/K212R for alpinetin formation. Apparent initial velocities were plotted against varying concentrations of pinocembrin (6.25, 15.625, 31, 62.5, 156, 312.5, and 625 µM), 50 µM purified StrAOMT variants, 2 mM SAM, 2 mM Mg^2+^ in Tri-HCL buffer (pH 7.5) at 37 °C. Data points represent mean ± SD, *n* = 3. Time-course data used to determine apparent initial velocities are shown in Figure S6

Based on the published crystal structures of StrAOMT in complex with SAH (PDB ID 8C9S) [19] and substrate-bound SafC (PDB ID 5LOG) [36], we performed docking experiments using pinocembrin as the ligand [34]. Ten poses were generated with scores ranging from 252.677 to 260.505, where lower scores indicate more favorable predicted interactions. The relatively narrow score range suggests the poses may be in a similar region of the binding site, but represent slightly different orientations or contact networks. After manually inspecting all docking poses, we first identified those that exhibited structural analogy to the experimental SafC structure and satisfied steric complementarity, and then selected from these the pose with the lowest score. In this selected pose, hydrogen bonds were observed between pinocembrin and Asp141, Asn168, Lys212, and Mg^2+^ (Fig. 4A). The distance between the side chain amine of Lys144 and the 5- hydroxy group of pinocembrin is 3.5 Å. The 5-hydroxyl group and the 3-carbonyl group of pinocembrin are likely to form a bidentate interaction with the Mg²⁺ ion, stabilizing the substrate within the active site.

**Fig. 5:**
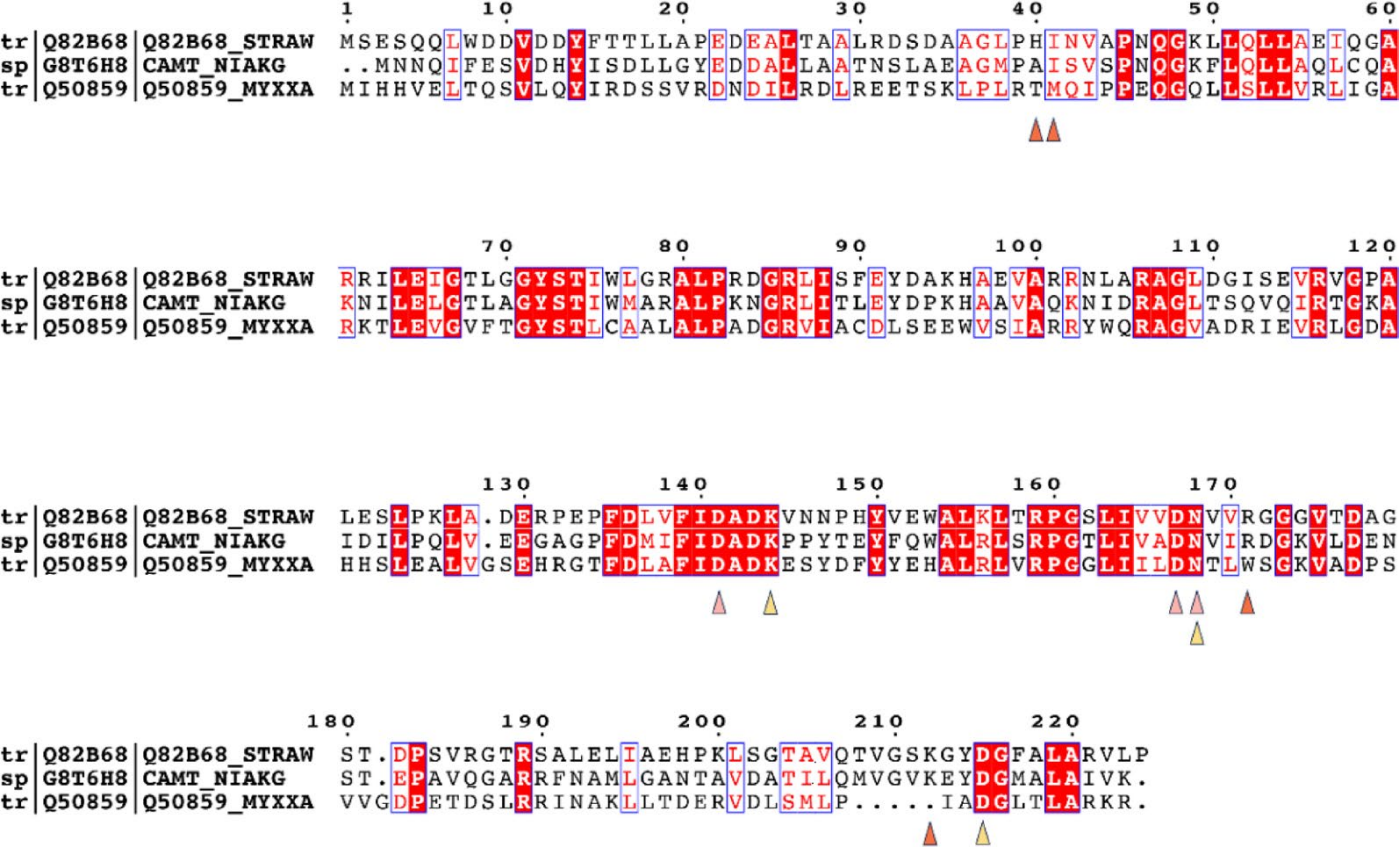
Protein sequence alignment of OMTs (1. O-methyltransferase from *Streptomyces avermitilis*, 2. O-methyltransferase from *Niastella koreensis*, and 3. O-methyltransferase from *Myxococcus xanthus*). Pink triangles indicate the metal-binding sites, yellow triangles indicate the catalytic triad, and orange triangles indicate the residues selected for directed evolution

Under the catalytic action of Lys144, the 5-hydroxyl group undergoes deprotonation, generating a phenolate anion. This deprotonated pinocembrin anion then acts as a nucleophile, attacking the methyl group of SAM in an SN_2_ mechanism, resulting in direct methyl transfer and the formation of the methylated product [49]. None of the docking poses shows an axial arrangement of the nucleophile with the methyl donor site of the cofactor, which would be indicative of the transition state arrangement. Repeating the docking with a modeled SAM-bound structure gave the same results (Figure S7A), indicating a re-orientation of the substrate and/or cofactor in the active site would be required for efficient catalysis. The final model of the substrate-bound StrAOMT was then exported for further analysis. Upon examining residues within 5 Å of pinocembrin, we identified 11 residues: H40, I41, D141, A142, D143, K144, D167, N168, R171, K212, and D215. Notably, D141, D167, and N168 form the metal-binding site in StrAOMT and K144, N168, and D215 the catalytic triad, which is strictly conserved in this OMT family. Next, we performed a protein sequence alignment of SafC (Uniprot accession: Q50859), StrAOMT (Uniprot accession: Q82B68), and NiaKOMT (Uniprot accession: G8T6H8) (Fig. 5). In addition to the metal binding and the catalytic residues, positions A142 and D143 were highly conserved among OMTs and were therefore excluded from further mutagenesis. Consequently, the positions H40, I41, R171, and K212 were selected for further analysis, and we performed site-saturation mutagenesis for these four positions individually using NNK degenerate codons (N = A/T/G/C; K = G/T), which encode all 20 amino acids.

The resulting mutant libraries were transformed into *E. coli* BL21 (DE3) and screened by evaluating ~ 100 transformants of each library. The screening led to an improved variant of StrAOMT K212R, which exhibited a 4.5-fold increase in alpinetin concentration compared to the wild-type enzyme (Supplementary Material 2). To further enhance the overall performance of StrAOMT, we employed an iterative saturation mutagenesis (ISM) strategy, using the best hits from the single-site libraries as templates and randomizing the other respective positions. Consequently, the libraries H40NNK/K212R, I41NNK/K212R, and R171NNK/K212R were constructed. Screening these libraries revealed that the best variant from each variant library were the combinations I41L/K212R, and R171W/K212R, resulting in a 1.6-fold and 2-fold increase in alpinetin concentration after 6 h compared to the StrAOMT K212R, respectively. However no improved variant was found in the H40NNK/K212R library (Supplementary Material 2). We then used StrAOMT I41L/K212R and R171W/K212R as templates to generate the triple mutant libraries StrAOMT I41L/R171NNK/K212R/, and I41NNK/R171W/K212R. Screening these mutants showed that the triple mutant StrAOMT I41L/R171W/K212R achieved a 1.8-fold increase in alpinetin concentration compared to the double mutant StrAOMT I41L/K212R and by 1.4-fold compared to StrAOMT R171W/K212R (Supplementary Material 2). We then purified various StrAOMT variants, including the wild-type, K212R, I41L/K212R, R171W/K212R, and I41L/R171W/K212R, for in vitro reactions. The results revealed significant increases in alpinetin production compared to the wild type: StrAOMT K212R, R171W/K212R, and I41L/R171W/K212R displayed a 68-fold, 50-fold, and 81-fold increase, respectively. StrAOMT I41L/K212R showed a more modest 3-fold increase (Fig. 4B). The results indicate that the K212R mutation has the strongest effect on the interaction with the substrate and/or the catalytic activity of the enzyme and that the I41L mutation is only beneficial in combination with the R171W mutation.

Intrigued by the observation that the triple mutant exhibited 81-fold increase in alpinetin titer, we performed steady-state kinetic assays for StrAOMT wild-type and StrAOMT I41L/R171W/K212R using SAM at a fixed concentration (2 mM) and pinocembrin at variable concentrations. Plotting the apparent initial reaction velocities at different pinocembrin concentrations allowed us to fit the data with the Michaelis−Menten equation (Figs. 4C, D, and S6). The k_cat_ of the triple mutant is 3.5-fold higher than that of the wild type, indicating enhanced catalytic efficiency of StrAOMT I41L/R171W/K212R. Additionally, the *K*_M_ of StrAOMT wild type is twice that of the StrAOMT triple mutant, indicating that the StrAOMT I41L/R171W/K212R variant has a higher affinity for pinocembrin compared to the wild type. Additional docking experiments with a modelled structure of the StrAOMT I41L/R171W/K212R variant does not provide deeper insight into the effect of the mutations on substrate binding or catalysis (Figure S7). It is, however, possible that the introduced arginine and tryptophane side chains engage in π-π interactions with the aromatic rings of the substrate and therefore stabilize a more favorable angle between the methyl donor and the nucleophile. Still, the affinity of the triple mutant for pinocembrin is rather low, which suggests that OMT may not work optimally in vivo because pinocembrin concentrations are too low.

### Production of alpinetin in resting cells expressing the enhanced O-methyltransferase variants

After identifying several mutants that showed improved affinity and catalytic activity towards pinocembrin compared to the wild type, we assessed their performances in the context of the flavonoid biosynthesis pathway. We generated the strains s11-s14 harboring these mutants, and carried out biotransformations in resting cells alongside s7 to assess whether the improved variants of StrAOMT enhance alpinetin production. The results showed that strain s7 produced approximately 0.13 µM alpinetin, while the highest alpinetin titer was achieved with strain s14 harboring the triple mutant of StrAOMT (0.96 µM), representing about a 7-fold increase. Still, the majority of the pinocembrin produced in this strain remains unmethylated, which is likely related to the moderate affinity of the OMT enzyme for pinocembrin. However, the poor in vivo performance cannot be fully explained by this effect.

**Fig. 6:**
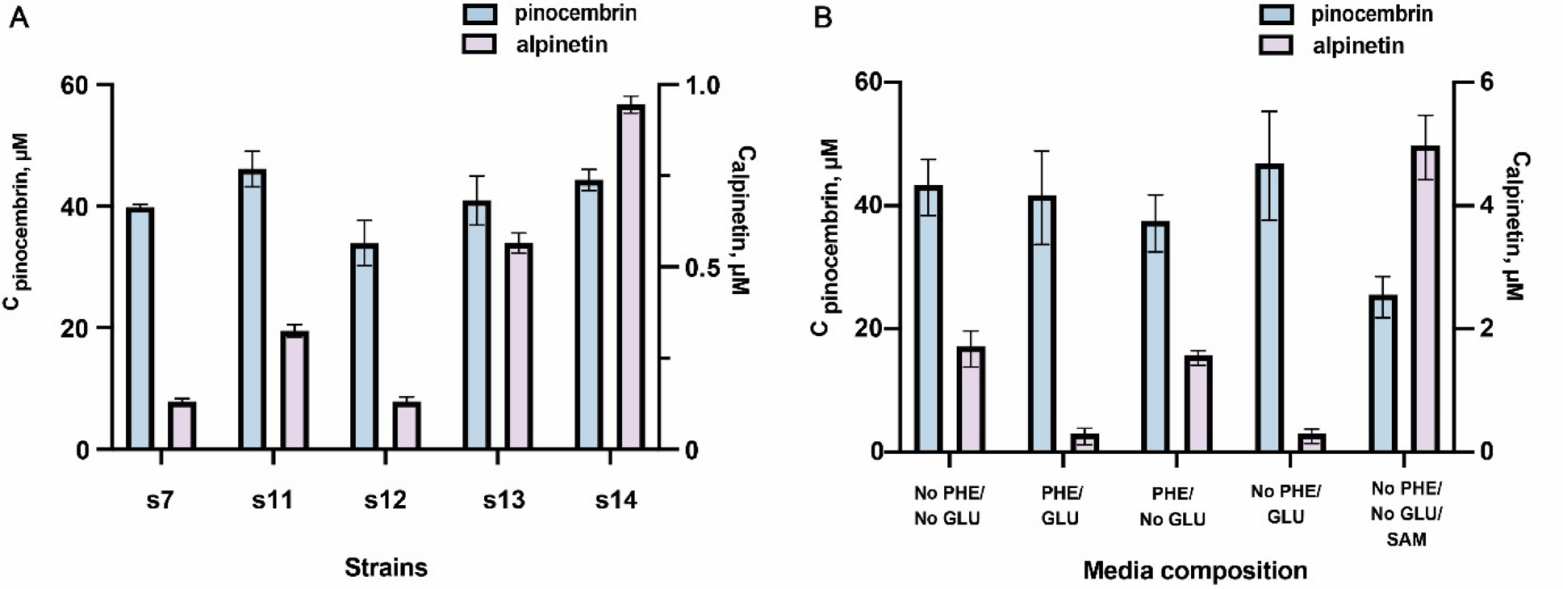
**A** Biotransformations of fed phenylalanine to alpinetin in resting *E. coli* BL21 (DE3) harboring the recombinant flavonoid biosynthesis pathways and StrAOMT variants. **B** Optimization of the media composition during the resting cell biotransformations with s14. Bars represent mean +/- SD, *n* = 3. Titers of pinocembrin (blue) and alpinetin (ashy purple) were determined after 36 h of incubation

To further rationalize our findings, we collected and analyzed extracts from the supernatant and the cell pellet separately and observed that - under the given extraction conditions – the majority of pinocembrin was present in the extract of the supernatant. We hypothesized that the intracellular concentration of pinocembrin at any given moment is too low for StrAOMT to catalyze the methylation efficiently. As a result, we decided to vary the media composition of the biotransformation step to assess whether supplementation of phenylalanine, glucose and extracellular SAM were beneficial to alpinetin production in the best-performing strain s14 (Fig. 6B). The results indicated that phenylalanine supplementation did not impact final alpinetin production, but rather reduced the amount of accumulated pinocembrin (Fig. 6). Glucose addition reduced the final titers of both alpinetin and pinocembrin. Supplementation with SAM increased the final alpinetin titer 2.9-fold, from 1.71 µM to 4.98 µM, in the absence of phenylalanine and glucose. Interestingly, we also detected naringenin (around 5 µM) and 5-methyl-naringenin, a 5-O-methylated flavonoid commonly co-occurring with alpinetin in *Alpinia* genus plants, [50] at concentrations below 1 µM. The production of 5-methyl-naringenin could be due to the promiscuity of XAL. In Indeed, a previous study showed that XAL from *Rhodotorula mucilaginosa* is capable of producing high concentrations of cinnamic acid from phenylalanine and low concentrations of *p*-coumaric acid from tyrosine [43]. *P*-coumaric acid can then enter the flavonoid biosynthetic pathway, leading to the production of naringenin, which can subsequently be methylated to form 5-methyl-naringenin. To confirm this hypothesis, we constructed the plasmid c15 lacking the gene encoding XAL and co-transformed it with c7, and c14 to generate strain s15. When fed with 0.1 mM cinnamic acid as substrate, the resting strain under optimal media conditions produced alpinetin at a concentration of 1.5 µM with no naringenin and 5-methyl-naringenin detected (SI Figure S8).

### Substrate scope of StrAOMT variants

**Fig. 7:**
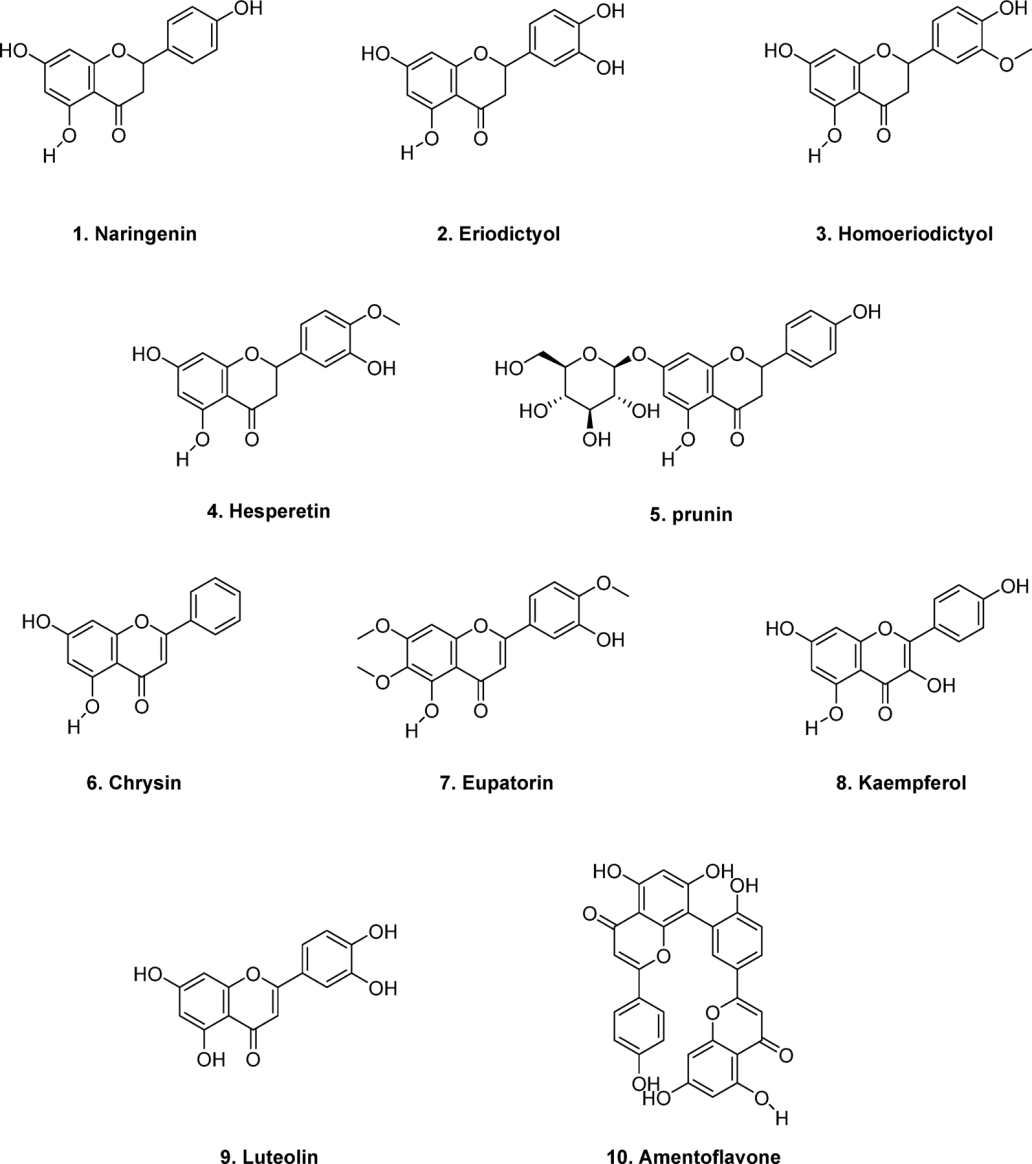
Substrate scope of the methylation reactions catalyzed by StrAOMT wildtype, StrAOMT K212R, and StrAOMT I41L/R171W/K212R. Reaction conditions: 25 mM Tris-HCl (pH 7.5), 2 mM MgCl₂, 1 mM SAM, 500 µM substrate, and 50 µM enzyme, in a final volume of 100 µL, with incubation at 37 °C for 12 h

Intrigued by the result that the triple mutant variant also produced 5-methyl-naringenin in whole cells, we explored how the substrate scope may have changed compared to our study from 2023 [19]. Thus, we performed in *vitro* experiments with the top two variants (StrAOMT K212R, and StrAOMT I41L/R171W/K212R) and StrAOMT wildtype on other flavonoids: naringenin (**1**), eriodictyol (**2**), homoeriodictyol (**3**), hesperetin (**4**), prunin (a flavanone glycoside, **5**), chrysin (**6**), eupatorin (a trimethoxyflavone, **7**), kaempferol (**8**), luteolin (**9**), and amentoflavone (**10**) (Fig. 7). Methylation products were identified based on characteristic shifts in retention times (RT) and an increase in *m/z* values by + 14 (single methylation) or + 28 (double methylation), relative to the corresponding substrates (Table 3, Figures S9-S11. Both low-resolution LC-MS and high-resolution tandem MS were used, with data compared against natural product databases and literature reports on O-methylated flavonoids [51]. In the absence of OMTs, only the unmodified substrate was detected. Using naringenin (**1**, *m/z* 273.0756, [M + H]^+^, RT = 5.62 min) as a substrate, StrAOMT variants produced a single monomethylated product (**1a**) with a retention time of 5.22 min, confirmed as naringenin 5-methyl ether based on a commercial reference standard. For eriodictyol (**2**, *m/z* 289.0706, [M + H]^+^, tR = 6.63 min), two monomethylated products were detected at 7.52 min and 7.65 min, identified as homoeriodictyol (**3**) and hesperetin (**4**), respectively. When homoeriodictyol (**3**, *m/z* 303.0863, [M + H]^+^, RT = 7.50 min) was used as a substrate, two monomethylated products (**3a** and **3b**) were detected at 6.20 min and 8.42 min. MS2 fragmentation analysis suggested that **3a** is methylated on ring A, and its earlier retention time compared to **3** indicates that it could be homoeriodictyol 5-methyl ether. The later-eluting product **3b** exhibited an MS2 fragment pattern consistent with methylation at the 4’-hydroxy group on ring B, identifying it as homoeriodictyol 4’-methyl ether (**3b**), commercially known as hesperetin 3’-methyl ether. For hesperetin (**4**, *m/z* 303.0863, [M + H]^+^, RT = 7.65 min), two monomethylated products were detected at 6.47 min and 8.42 min (**4a** and **4b**, respectively). MS2 analysis of **4a** suggests methylation on ring A, with its earlier retention time suggesting that it is hesperetin 5-methyl ether. The later-eluting product **4b** has the identical RT and M2 fragmentation pattern as **3b**, again supporting its identification as hesperetin 3’-methyl ether. When prunin (**5**, *m/z* 435.1288, [M + H]^+^, RT = 5.17 min) was used as a substrate, two monomethylated products (**5a** and **5b**) were detected at 4.77 min and 5.59 min. MS2 analysis indicated that **5a** is methylated on ring A at the 5-hydroxy position, identifying it as prunin 5-methyl ether. In contrast, **5b** exhibits a fragmentation pattern consistent with methylation on ring B, corresponding to prunin 4’-methyl ether (commercially known as isosakuranin). For compounds **6**–**10**, a normalized collision energy (NCE) of 35 did not yield effective fragmentation. Therefore, a combination of 30, 40, and 55 NCE was used. For chrysin (**6**, *m/z* 255.0654, [M + H]^+^, RT = 6.09 min), a single monomethylated product (**6a**, RT = 5.66 min) was detected. While MS2 analysis did not provide definitive structural information, its earlier retention time suggests it is likely chrysin 5-methyl ether [47, 52]. When eupatorin (**7**, *m/z* 345.0971, [M + H]^+^, RT = 5.96 min) was used as a substrate, two monomethylated products (**7a** and **7b**) were detected at 5.72 min and 6.21 min. Based on previous reports on HPLC separation, **7a** was identified as eupatorin 5-methyl ether, while **7b** likely corresponds to eupatorin 3’-methyl ether (commercially known as 5-desmethylsinensetin) [53]. For kaempferol (**8**, *m/z* 287.0550, [M + H]^+^, RT = 5.66 min), two monomethylated products (**8a** and **8b**) were detected at 5.37 min and 5.74 min, along with one di-methylated product (**8c**) at 5.32 min. Based on literature, kaempferol 3- and 5-methyl derivatives are prone to CH₄ loss under tandem MS due to their spatial proximity to the C4-keto group, forming a fragment with *m/z* 285 [M + H]^+^. The detection of this fragment in both **8a** and **8b** and their relative retention times suggests that they correspond to kaempferol 5-methyl ether and kaempferol 3-methyl ether, respectively [51]. The di-methylated product (**8c**) exhibited a distinctive *m/z* 299.0550 fragment, likely resulting from steric and electronic effects that hinder the characteristic Retro-Diels-Alder fragmentation of flavonoids, favoring CH₄ loss instead [51]. Consequently, we propose it to be kaempferol 3,5-dimethyl ether, further supported by the RT, which is even smaller than that of **8a**. Luteolin (**9**, *m/z* 287.0549, [M + H]^+^, RT = 5.44 min) yielded a monomethylated product (**9a**, RT = 5.67 min), closely matching 5,7-dihydroxy-2-(4-hydroxy-3-methoxyphenyl) chromen-4-one based on the GNPS Library Spectrum CCMSLIB00000079846. Additionally, a di-methylated product (**9b**, RT = 5.29 min) was detected, though the specific methylation sites could not be determined. For amentoflavone (**10**, *m/z* 539.0983, [M + H]^+^, RT = 5.79 min), one monomethylated product (RT = 5.54 min) was detected. MS2 comparison with the GNPS database showed a strong match with amentoflavone 4’-methyl ether (Spectrum ID: CCMSLIB00005724613).

After putatively identifying the methylated products, we compared the three enzyme variants by analyzing the apparent product peak areas obtained from the chromatograms. Owing to low product yields and the lack of commercially available standards for some methylated products, absolute quantification was not performed; instead, relative peak areas were used to compare the activities of the different variants (Figure S11). This analysis indicates that the triple mutant exhibits enhanced activity towards the formation of naringenin 5-methyl ether, homoeriodictyol 5-methyl ether, hesperetin 5-methyl ether, prunin 5-methyl ether, eupatorin 5-methyl ether, amentoflavone 4’-methyl ether, and luteolin dimethyl ether compared to wild type and double mutant. Notably, it showed a high selectivity for hesperetin 5’-methyl ether formation (> 99%). Meanwhile, the double mutant demonstrated increased activity towards chrysin 5-methyl ether and kaempferol dimethyl ether compared to wild type and triple mutant. This suggests that our mutagenesis campaign did influence the regioselectivity of StrAOMT but further engineering is necessary to further enhance the substrate conversion and regioselectivity.

**Table 3:** Characteristics of the major peaks detected via HPLC-MS and high-resolution tandem MS in the extracts of enzymatic reactions of the StrAOMT variants

Peak/compound no.	RT^a^ [min]	Molecular formula	[M+H]^+^ (error [ppm])	MS/MS fragment ions		Best match based on literature
				[M+H]+[m/z]	Intensity %^b^	
**1**	5.62	C_15_H_12_O_5_	273,0757	153,0183	100	naringenin
			(2.2)	147,0441	75	
				273,0757	65	
				274,0792	10	
				154,0217	9	
**1a**	5.22	C_16_H_14_O_5_	287,0913	167,0341	100	naringenin 5-methyl ether
			(2.26)	147,0441	12	
				168,0373	10	
				287,0914	8	
**2**	6.63	C_15_H_12_O_6_	289,0706	163,039	100	eriodictyol
			(2.13)	153,0182	74	
				289,0704	45	
				179,0338	12	
**3**	7.52	C_16_H_14_O_6_	303,0865	153,0184	100	homoeriodictyol
			(1.2)	177,0549	100	
				303,0865	18	
				179,034	16	
**3a**	6.20	C_17_H_16_O_6_	317,1016	167,034	100	homoeriodictyol 5-methyl ether
			(2.89)	177,0547	25	
				145,0284	8	
				317,1022	5	
**3b/4b**	8.42	C_17_H_16_O_6_	317,102	153,0182	100	hesperetin 3’-methyl ether
			(1.62)	191,0703	87	
				171,0288	18	
				179,0338	16	
**4**	7.65	C_16_H_14_O_6_	303,0863	177,0547	100	hesperetin
			(1.86)	153,0182	65	
				303,0862	25	
**4a**	6.47	C_17_H_16_O_6_	317,1018	167,034	100	hesperetin 5-methyl ether
			(2.25)	177,0548	40	
**5**	5.17	C_21_H_22_O_10_	435,1288	273,0759	100	prunin
			(0.75)	153,0184	58	
				147,0442	43	
**5a**	4.77	C_22_H_24_O_10_	449,1446	287,0912	100	prunin 5-methyl ether
			(0.39)	167,034	87	
				288,0947	15	
**5b**	5.59	C_22_H_24_O_10_	449,1447	287,0914	100	prunin 4-methyl ether
			(0.17)	153,0183	30	(Isosakuranin)
				161.0598 288.0946	30 17	
**6**	6.09	C_15_H_10_O_4_	255,0654	255,0655	100	chrysin
			(1.31)	153,0186	50	
				68,9972	37	
				103,0543	32	
				129,0337	18	
**6a**	5.66	C_16_H_12_O_4_	269,0808	124,0156	100	chrysin 5-methyl ether
			(2.17)	226,0626	95	
				254,0574	34	
				68,9971	33	
				170,0211	20	
				152,0106	20	
**7**	5.96	C_18_H_16_O_7_	345,097	284,0675	100	eupatorin
			(1.25)	108,0203	80	
				148,0517	60	
				312,0623	50	
				185,0595	35	
**7a**	5.72	C_19_H_18_O_7_	359,1125	153,0182	100	eupatorine 5-methyl ether
			(1.62)	255,0648	35	
				329,0655	32	
				151,0390	30	
				227,0703	25	
**7b**	6.21	C_19_H_18_O_7_	359,1125	298,0833	100	eupatorin 3’-methyl ether (5-desmethylsinensetin)
			(1.62)	162,0676	70	
				326,0783	70	
				108,0205	45	
				359,1125	35	
**8**	5.66	C_15_H_10_O_6_	287,0550	153,0181	100	kaempferol
			(1.97)	287,0545	95	
				121,0283	70	
				68,9970	65	
				165,0181	25	
**8a**	5.37	C_16_H_12_O_6_	301,0706	286,0468	100	kaempferol 5-methyl ether
			(2.04)	285,0394	75	
				121,0285	40	
				301,0706	35	
				258,0523	25	
**8b**	5.74	C_16_H_12_O_6_	301,0704	121,0284	100	kaempferol 3-methyl ether
			(2.71)	286,0463	42	
				285,0463	30	
				94,0413	30	
				68,9971	28	
**8c**	5.32	C_17_H_14_O_6_	315,0862	254,0573	100	kaempferol-3.5-di-methyl ether
			(2.11)	282,0518	32	
				198,0674	28	
				299,055	28	
				197,0598	25	
**9**	5.44	C_15_H_10_O_6_	287,0554	287,0548	100	luteolin
			(0.57)	153,0183	55	
				68,9971	25	
				89,0384	22	
				135,0441	20	
**9a**	5.67	C_16_H_12_O_6_	301,0706	258,0523	100	luteolin methyl ether
			(2.04)	286,0472	60	
				153,0183	49	
				229,0497	37	
				301,0706	16	
**9b**	5.29	C_17_H_14_O_6_	315,0863	300,0627	100	luteolin dimethyl ether
			(1.79)	229,0495	90	
				272,0678	42	
				315,0862	30	
				257,0444	30	
**10**	5.79	C_30_H_18_O_10_	539,0983	69,9828	100	amentoflavone
			(0.88)	121,0287	38	
				96,2029	30	
				153,0181	28	
				255,4802	20	
**10a**	5.54	C_31_H_20_O_10_	553,114	121,0286	100	amentoflavone 4’-methyl ether
			(0.95)	553,1172	38	
				391,0847	12	
				163,0393	12	
				521,0900	10	

^a^Retention times as shown in Figure S9 with low-resolution HPLC-MS data. ^b^Peak height relative to peak height of main fragment

## Discussion

Alpinetin is a naturally occurring 5-O-methylated flavonoid with diverse bioactivities, widely found in medicinal plants such as *Mikania micrantha Kunth*, *Combretum albopunctatum Suess*., *Dalbergia odorifera* T.C.Chen, *Scutellaria indica* L., *Persicaria ferruginea*, and *Alpinia hainanensis* K.Schum [14, 16]. Among these, the *Zingiberaceae* family has the highest levels of alpinetin, which is particularly abundant in its seeds and rhizomes, containing approximately 5 mg per gram of dry biomass. Despite its low abundance in plants, the extraction process relies on solvents such as methanol, ethanol, dichloromethane, and chloroform, which pose environmental and safety concerns. While significant advances have been made in the biosynthesis of pinocembrin, alpinetin biosynthesis remains largely underexplored [38–42].

In this study, we successfully achieved the *de novo* biosynthesis of alpinetin in *E. coli* for the first time by choice of pathway enzymes, optimizing plasmid configurations, medium composition, and fermentation strategies. Additionally, we enhanced the activity of StrAOMT through directed evolution, resulting in the StrAOMT I41L/R171W/K212R triple mutant. This variant demonstrated an 81-fold increase in alpinetin production in vitro compared to the wild-type enzyme. Michaelis-Menten analysis further revealed that the mutant enzyme had a higher turnover number and improved affinity for pinocembrin. Using this improved enzyme, we achieved a final alpinetin titer of approximately 4.98 µM. Interestingly, StrAOMT variants exhibited O-methylation activity toward a broad range of flavonoids, producing valuable 5-O-methylated compounds such as naringenin 5-methyl ether, homoeriodictyol 5-methyl ether, hesperetin 5-methyl ether, prunin 5-methyl ether, chrysin 5-methyl ether, eupatorine 5-methyl ether, and kaempferol 5-methyl ether. Additionally, they produced commercially valuable flavonoids such as isosakuranin and 5-desmethylsinensetin, whose biosynthesis had not been previously reported [53, 54]. Moreover, the StrAOMT I41L/R171W/K212R triple mutant displayed altered regioselectivity toward hesperetin, homoeriodictyol 5-methyl ether, and hesperetin 5-methyl ether formation. These findings highlight the potential of StrAOMT variants for the selective biosynthesis of these valuable O-methylated flavonoids in the future. Recently, the *de novo* biosynthesis of the prenylated flavonoid xanthohumol was reported, with the final titers of xanthohumol and desmethylxanthohumol indicating that the methylation step needs further optimization. Our StrAOMT variants demonstrate significant potential for efficiently methylating this compound [55].

The final alpinetin titer we achieved remains significantly below market demand. Swapping StrAOMT from the high–copy number plasmid pRSFDuet-1 to the moderate–copy number plasmid pCDFDuet-1 enabled production of alpinetin, as well as increased levels of cinnamic acid and pinocembrin, suggesting a metabolic burden in the alpinetin biosynthetic pathway. In the future, balancing the expression of pathway enzymes should be considered, for example by using plasmids with different promoters or Gram-negative replicons to fine-tune gene expression in the alpinetin pathway. Future efforts should also focus on improving the spatial organization of the pathway, for instance by co‑localizing pinocembrin and StrAOMT variants within the same cellular compartment, such as through surface display of the OMT[56–58]. Since we did not determine the kinetic parameters of the upstream enzymes in the cascade – in particular the pinocembrin‑producing CHS – we cannot unambiguously conclude whether the turnover number of the OMT is ultimately rate‑limiting. However, when comparing the affinity of StrAOMT for pinocembrin with the highest pinocembrin titers obtained in this study, it becomes evident that the OMT operates under sub‑saturating substrate concentrations and therefore cannot reach its maximal catalytic velocity, even if all pinocembrin were retained intracellularly. This suggests that any loss of pinocembrin from the cytosol, for example through export, further reduces the effective substrate availability for the OMT. To address this limitation, enzyme tethering or scaffolding strategies that physically colocalize CHS and OMT could enhance metabolic channeling and reduce the probability of pinocembrin diffusing away before methylation. Moreover, the export mechanism of flavonoids in *E. coli* is not well defined and likely involves multiple unspecific efflux transporters [59–61]. Consequently, suppressing pinocembrin export would require systematic deletion or downregulation of several candidate transporters, which may compromise strain robustness and viability. Alternatively, using a different host such as yeast, which offers compartmentalization and may reduce precursor efflux, could be beneficial for improving methylation efficiency [62–64]. Improving the availability of co-substrates for both the chalcone synthase (malonyl-CoA) [65] and OMT (SAM) [36, 66] should also further enhance the efficiency of the pathway. Although an 81-fold increase in alpinetin production in vitro was achieved, the catalytic turnover of the triple mutant is still relatively low and could be further improved by semi-rational engineering. Alternatively, exploring OMTs from *Alpinia* species may offer additional advantages. For alpinetin biosynthesis from phenylalanine, selecting an aromatic amino acid ammonia lyase with low activity toward *p*-coumaric acid but high specificity for phenylalanine would be beneficial. Potential candidates include PpPAL from *Physcomitrella patens subsp. patens*, DdPAL from *Dictyostelium discoideum*, and BlPAL from *Brevibacillus laterosporus *[43].

## Conclusions

In summary, we achieved *de novo* biosynthesis of alpinetin from phenylalanine in *E. coli* for the first time. The engineered StrAOMT variants hold great potential for the selective biosynthesis of valuable O-methylated flavonoids in the future.

## Supplementary Information

Supplementary Material 1.

Supplementary Material 2.

## Data Availability

All data supporting the findings of this study are included in the manuscript or the supporting information, or can be retrieved upon reasonable request from the corresponding author.
