# Supplementary material for: De novo biosynthesis of alpinetin enhanced by directed evolution of 5-O-methyltransferase
Source: Microb Cell Fact. 2026 Apr 6;25:134. doi: 10.1186/s12934-026-02996-x (PMC13214397; doi:10.1186/s12934-026-02996-x)
Supplement: Supplementary file 2 — Supplementary Material 2. [file 12934_2026_2996_MOESM2_ESM.docx]

# Supporting Information

# *De novo* biosynthesis of alpinetin enhanced by directed evolution of 5-O-methyltransferase

Bo Peng^a^, Ziwei Wang^a^, Lili Zhang^a^, Matthew R. Groves^a,b^, Kristina Haslinger^a^

a. Department of Chemical and Pharmaceutical Biology, University of Groningen, The Netherlands

b. Genomics for Health in Africa (GHA), Africa-Europe Cluster of Research Excellence (CoRE).

Contents

[Sequences of synthetic genes 2](#_Toc202772632)

[Supporting Tables 4](#_Toc202772633)

[Supporting Figures 5](#_Toc202772634)

## Sequences of synthetic genes

**RmXAL (*Rhodotorula mucilaginosa*, GenBank accession number KR095285)**

ATGGCACCGAGCGTTGATAGCATTGCAACCAGCGTTGCAAATAGCCTGAGCAATGGTCTGCATGCCGCAGCAGCAGCAAATGGTGGTGATGTTCACAAAAAAACCGCAGGCGCAGGTAGCCTGCTGCCGACCACCGAAACCACCCAGCTGGATATTGTTGAACGTATTCTGGCAGATGCCGGTGCAACCGATCAGATTAAACTGGATGGTTATACCCTGACCCTGGGTGATGTTGTTGGTGCAGCACGTCGTGGTCGTAGCGTTAAAGTTGCAGATAGTCCGCATATTCGCGAAAAAATTGATGCCAGCGTTGAATTTCTGCGTACCCAACTGGATAATAGCGTTTATGGTGTTACCACCGGTTTTGGTGGTAGCGCAGATACCCGTACCGAAGATGCAATTAGCCTGCAGAAAGCACTGCTGGAACATCAGCTGTGTGGTGTTCTGCCGACCTCAATGGATGGTTTTGCACTGGGTCGTGGTCTGGAAAATAGTCTGCCGCTGGAAGTTGTTCGTGGTGCAATGACCATTCGTGTTAATAGTCTGACCCGTGGTCATAGTGCAGTTCGTATTGTTGTTCTGGAAGCACTGACCAATTTTCTGAATCATGGTATTACCCCGATTGTTCCGCTGCGTGGCACCATTAGCGCAAGCGGTGATCTGAGTCCGCTGAGCTATATTGCAGCAAGCATTACCGGTCATCCGGATAGCAAAGTTCATGTTGATGGCAAAATTATGAGCGCACAAGAAGCAATTGCACTGAAAGGTCTGCAGCCGGTTGTGCTGGGTCCGAAAGAAGGTCTGGGTCTGGTTAATGGCACCGCAGTTAGCGCCAGCATGGCAACCCTGGCACTGACCGATGCACATGTTCTGAGCCTGCTGGCACAGGCCCTGACCGCACTGACAGTTGAAGCAATGGTTGGTCATGCAGGTAGCTTTCATCCGTTTCTGCATGATGTTACCCGTCCGCATCCGACCCAGATTGAAGTTGCACGTAATATTCGTACCCTGCTGGAAGGTAGCAAATATGCAGTTCATCATGAAACCGAGGTGAAAGTGAAAGATGATGAAGGTATTCTGCGTCAGGATCGTTATCCGCTGCGCTGTAGTCCGCAGTGGCTGGGTCCTCTGGTTAGCGATATGATTCATGCACATGCAGTGCTGAGCCTGGAAGCAGGTCAGAGCACCACCGATAATCCGCTGATTGATCTGGAAAACAAAATGACCCATCATGGTGGTGCATTTATGGCAAGCAGCGTTGGCAATACCATGGAAAAAACCCGTCTGGCAGTTGCACTGATGGGTAAAGTTAGTTTTACCCAGCTGACCGAAATGCTGAATGCAGGTATGAATCGTGCACTGCCGAGCTGTCTGGCAGCAGAAGATCCGAGCCTGAGTTATCATTGTAAAGGTCTGGATATCGCAGCAGCCGCATATACCAGCGAACTGGGTCATCTGGCAAATCCGGTTAGCACCCATGTTCAGCCTGCCGAAATGGGTAATCAGGCAATTAATTCACTGGCCCTGATTAGCGCACGTCGTACAGCCGAAGCAAATGATGTTCTGTCACTGCTGCTGGCAACCCATCTGTATTGTGTGCTGCAGGCAGTTGATCTGCGTGCAATGGAATTTGAACATACCAAAGCATTTGAACCGATGGTTACAGAACTGCTGAAACAGCATTTTGGTGCACTGGCAACCGCAGAAGTTGAAGATAAAGTTCGTAAAAGCATCTACAAACGCCTGCAACAGAACAATAGCTACGATCTGGAACAGCGCTGGCATGATACCTTTAGCGTTGCCACCGGTGCAGTTGTTGAAGCACTGGCAGGTCAAGAAGTTAGCCTGGCAAGCCTGAATGCATGGAAAGTTGCATGTGCCGAAAAAGCCATTGCCCTGACCCGTAGCGTTCGTGATAGCTTTTGGGCAGCACCGAGCAGCAGCTCACCGGCACTGAAATATCTGTCACCGCGTACCCGTGTTCTGTATAGCTTTGTTCGTGAAGAAGTTGGCGTTAAAGCCCGTCGCGGTGATGTTTATCTGGGTAAACAAGAAGTGACCATTGGTACAAATGTGAGCCGTATTTATGAAGCCATTAAAAGCGGTTGTATTGCACCGGTTCTGGTTAAAATGATGGCCAAGCTTGCGGCCGCATAA

**Gm4CL (*Glycine max*, GenBank accession number X69955)**

ATGATTACCCTGGCACCGAGTCTTGATACCCCGAAAACCGATCAGAATCAGGTGAGCGATCCGCAGACCAGCCATGTGTTTAAATCGAAACTGCCGGATATCCCGATTAGCAACCATCTGCCGCTGCACAGCTACTGCTTCCAGAACCTGAGCCAGTTTGCCCATCGCCCGTGCCTGATTGTGGGTCCGGCCAGCAAAACCTTCACCTATGCGGATACCCACCTGATTAGCTCAAAAATTGCGGCGGGTCTGAGCAACCTTGGCATCCTGAAAGGCGATGTGGTGATGATTCTGCTGCAGAATAGCGCGGATTTCGTGTTTTCCTTTCTGGCGATTAGCATGATTGGCGCGGTCGCGACCACCGCGAATCCGTTTTACACCGCGCCGGAAATTTTTAAACAGTTTACGGTGAGCAAAGCCAAACTGATTATCACCCAGGCGATGTATGTGGATAAACTGCGCAATCACGATGGAGCCAAACTGGGCGAAGATTTTAAAGTTGTGACCGTGGATGATCCGCCGGAAAACTGCCTGCATTTTAGCGTTCTGAGCGAAGCCAACGAAAGCGATGTGCCGGAAGTTGAAATTCATCCGGATGATGCGGTCGCCATGCCGTTCAGCAGCGGTACCACCGGTCTGCCGAAAGGCGTGATTCTGACCCATAAAAGCCTGACCACGAGCGTGGCCCAGCAGGTGGATGGCGAAAACCCGAACCTGTATCTGACCACCGAAGATGTTCTGCTGTGTGTACTGCCGCTGTTTCATATTTTCAGCTTGAACAGCGTGCTGCTGTGTGCGCTGCGCGCGGGCAGTGCGGTGCTGCTGATGCAGAAATTCGAAATTGGCACCCTGCTGGAACTGATTCAGCGTCATCGTGTCAGCGTTGCAATGGTTGTGCCGCCGCTGGTTCTGGCGCTGGCCAAAAATCCGATGGTGGCGGATTTTGACCTGAGCAGCATTCGCCTGGTGCTGTCGGGCGCGGCACCGCTGGGCAAAGAACTGGAAGAGGCACTGCGTAATCGTATGCCGCAGGCGGTTCTGGGCCAGGGCTACGGCATGACCGAAGCGGGCCCGGTGCTGAGCATGTGTCTGGGCTTCGCCAAACAGCCGTTTCAGACCAAAAGCGGCAGCTGCGGCACCGTGGTGCGCAATGCCGAACTGAAAGTGGTGGATCCGGAAACCGGCCGCAGCCTGGGCTATAACCAGCCGGGCGAAATTTGTATTCGCGGCCAGCAAATTATGAAAGGCTATCTGAATGACGAAGCCGCAACCGCCTCAACCATTGATAGCGAAGGCTGGCTGCATACCGGCGATGTGGGCTATGTTGATGATGATGATGAAATTTTTATTGTGGATCGCGTGAAAGAACTGATTAAATATAAAGGCTTTCAGGTGCCGCCGGCGGAACTGGAAGGCCTGCTGGTGAGCCATCCGAGCATTGCGGATGCGGCGGTGGTGCCGCAGAAAGATGTGGCGGCCGGTGAAGTGCCGGTGGCGTTTGTGGTGCGCAGCAACGGTTTTGATCTGACCGAAGAAGCCGTAAAAGAATTTATTGCAAAACAGGTAGTGTTCTATAAACGCCTGCATAAAGTGTATTTTGTTCATGCCATTCCGAAAAGCCCGAGCGGCAAAATTCTGCGCAAAGATCTGCGTGCGAAACTGGAAACCGCCGCGACCCAGACCCCGTAA

**CsCHS (*Camellia sinensis*, GenBank accession number D26593):**

ATGGTGACCGTGGAAGATATTCGCCGTGCGCAGCGCGCCGAAGGCCCGGCGACCGTTATGGCGATTGGCACGGCGACCCCGCCGAATTGCGTGGATCAGAGCACCTACCCGGATTATTACTTTCGCATTACCAACAGCGAACACAAAGCGGAACTGAAAGAAAAATTTAAACGCATGTGCGATAAAAGCATGATTAAAAAACGTTATATGTATCTGACCGAAGAAATTCTGAAAGAAAACCCGCAGGTGTGTGAATATATGGCCCCGAGTCTGGATGCCCGCCAGGATATGGTGGTCGTGGAAGTGCCGAAACTGGGCAAAGAAGCCGCGACGAAAGCCATCAAAGAATGGGGTCAGCCGAAATCTAAAATTACGCACCTGGTTTTTTGTACCACCTCAGGCGTTGATATGCCGGGCGCGGATTACCAGCTGACCAAACTGCTGGGTCTGCGTCCGTCTGTGAAACGCCTGATGATGTATCAGCAGGGCTGCTTCGCGGGCGGCACCGTGCTGCGTCTGGCGAAAGATCTGGCGGAAAACAATAAAGGCGCCCGCGTGCTGGTGGTGTGCAGCGAAATTACCGCGGTGACCTTTCGTGGCCCGAGCGATACCCACCTGGATAGCCTGGTGGGTCAGGCGCTGTTTGGTGACGGCGCCGCGGCGATTATTGTTGGCAGCGATCCGATTCCGGAAGTGGAAAAACCGCTGTTCGAACTGGTGTCAGCGGCGCAGACCATTCTGCCGGATAGCGATGGCGCGATCGATGGTCATCTGCGCGAAGTGGGCCTGACCTTTCACCTGCTGAAAGATGTTCCGGGCCTGATTAGCAAAAACATTGAAAAAAGCCTGGCGGAAGCGTTCCAGCCGCTGGGCATCAGCGATTGGAACAGCCTTTTCTGGATTGCGCATCCGGGTGGCCCGGCGATTCTGGATCAGGTGGAACTGAAACTGGGCTTAAAAGAAGAAAAACTGCGCGCGACTCGCCACGTGCTGAGCGAATATGGCAACATGAGCAGCGCCTGCGTGCTGTTCATTCTGGATGAAATGCGCAAAAAAAGCGCAGCCGATGGCCTGAAAACCACGGGCGAAGGCCTGGAATGGGGCGTGCTGTTTGGCTTTGGCCCGGGCCTGACGGTGGAAACCGTGGTGCTGCATAGCCTGAGCACCTAA

## Supporting Tables

**Table S1**. Primers used in this study.

| **Primers** | **Primer sequences** |
| --- | --- |
| BPE_41NNK_FP | ggggccacattmnnatgaggcagaccggctg |
| BPE_41NNK_RP | gtctgcctcatnnkaatgtggccccgaacca |
| BPE_42NNK_FP | ggccacmnntatatgaggcagaccggctgc |
| BPE_42NNK_RP | ggtctgcctcatatannkgtggccccgaac |
| BPE_171NNK_FP | gtaactcccccaccmnngactacattgtctacgacga |
| BPE_171NNK_RP | tagacaatgtagtcnnkggtgggggagttacagatgc |
| BPE_212NNK_FP | aaccgtcgtaaccmnnggaacccaccgtctgaacc |
| BPE_212NNK_RP | gacggtgggttccnnkggttacgacggttttgctt |

## Supporting Figures


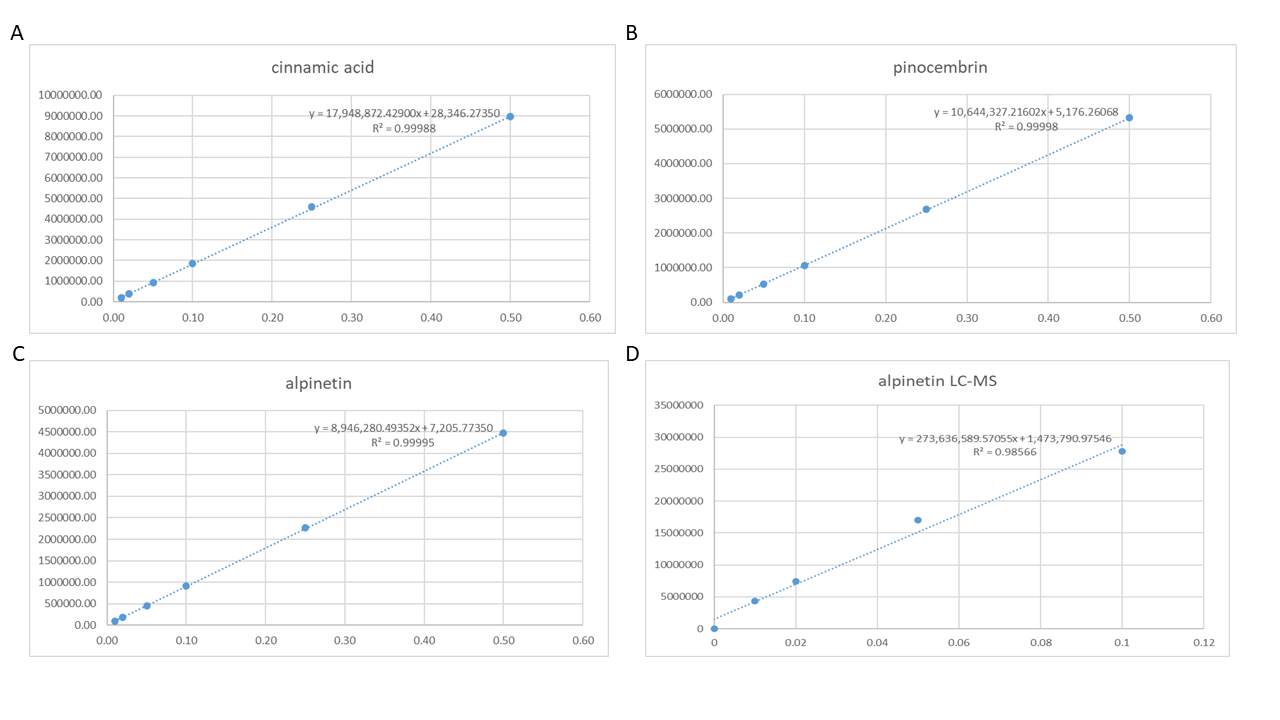


**Figure S1**. Calibration plots of cinnamic acid (A), pinocembrin (B), and alpinetin (C) dissolved in DMSO and analyzed by HPLC, and alpinetin (D) dissolved in methanol and analyzed by LC-MS. For HPLC analysis, the compounds were detected at 288 nm, with a calibration curve ranging from 0.01 mM to 0.5 mM. For LC-MS analysis, alpinetin was detected in positive ion mode, with a calibration curve ranging from 0 to 0.1 mM.


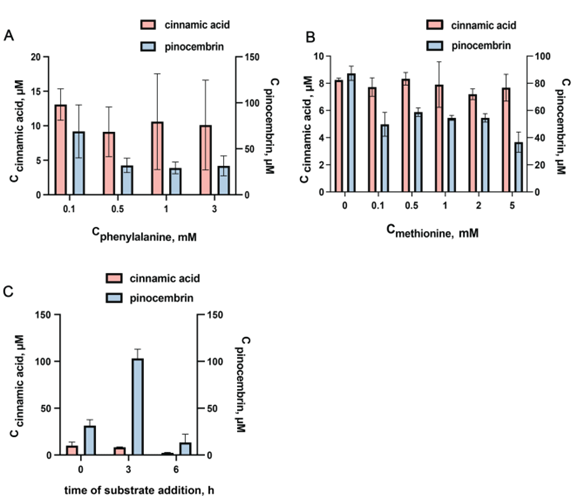


**Figure S2**. Fermentation of *E. coli* MG1655 (DE3) harboring the flavonoid biosynthesis pathway. Titers of cinnamic acid (pink, left axis) and pinocembrin (blue, right axis) were determined 48 h after induction of enzyme expression. A) Cinnamic acid and pinocembrin concentration upon feeding with different concentration of phenylalanine. B) Cinnamic acid and pinocembrin concentration upon feeding with different concentration of methionine. A) Cinnamic acid and pinocembrin concentration with varying substrate addition time. (bars represent mean +/-SD, n=3)

**Figure S3**. Biotransformation of fed phenylalanine (1 mM) in resting *E. coli* BL21 (DE3) strains harboring the flavonoid biosynthesis pathway and empty pRSFDuet-1 or pCDFDuet-1. Bars represent mean +/- SD, n=3.


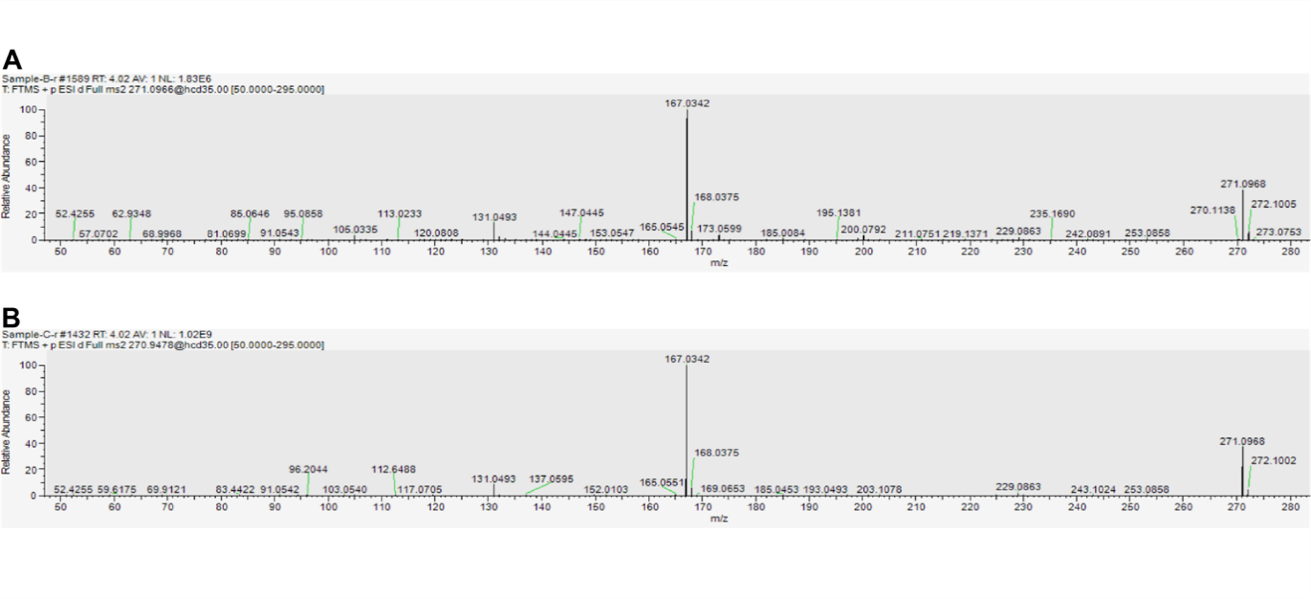


**Figure S4**. Product ion mass spectra (MS2) in high-resolution tandem MS of: A) alpinetin extracted from the S7 fermentation culture (m/z 271.0968, RT=4.02 min), B) commercial standard of alpinetin (m/z 271.0968, RT=4.02 min).


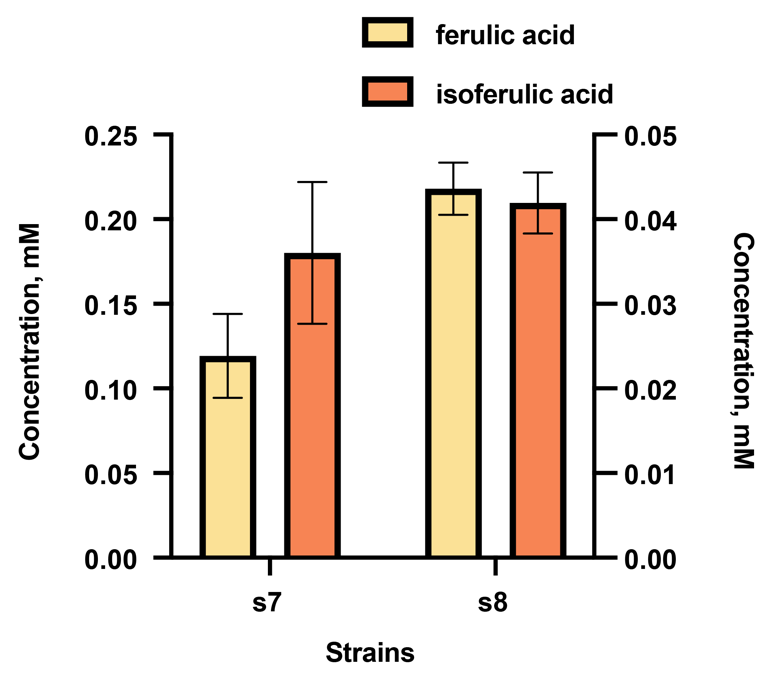


**Figure S5**. Biotransformation of fed caffeic acid (1 mM) in resting *E. coli* BL21 (DE3) strains harboring the flavonoid biosynthesis pathway. Bars represent mean +/- SD, n=3.


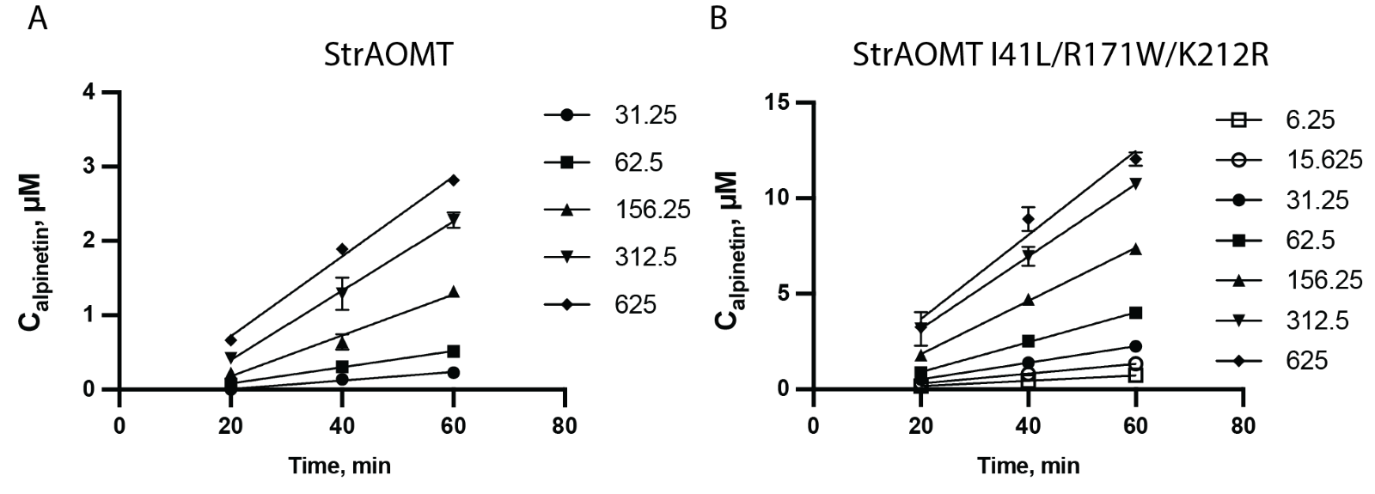


**Figure S6.** Time progress curves underlying the steady-state kinetics analysis. Alpinetin concentration obtained in *in vitro* turnovers catalyzed by A) StrAOMT wild type and B) StrAOMT I41L/R171W/K212R in the presence of varying concentrations of pinocembrin (6.25, 15.625, 31.25, 62.5, 156.25, 312.5, and 625 µM) and a fixed concentration of SAM (1 mM). Data points represent mean +/- SD, n=3, line represents linear regression to determine the apparent initial velocities for each substrate concentration. Replots of initial velocities are shown in Figure 4C and D.


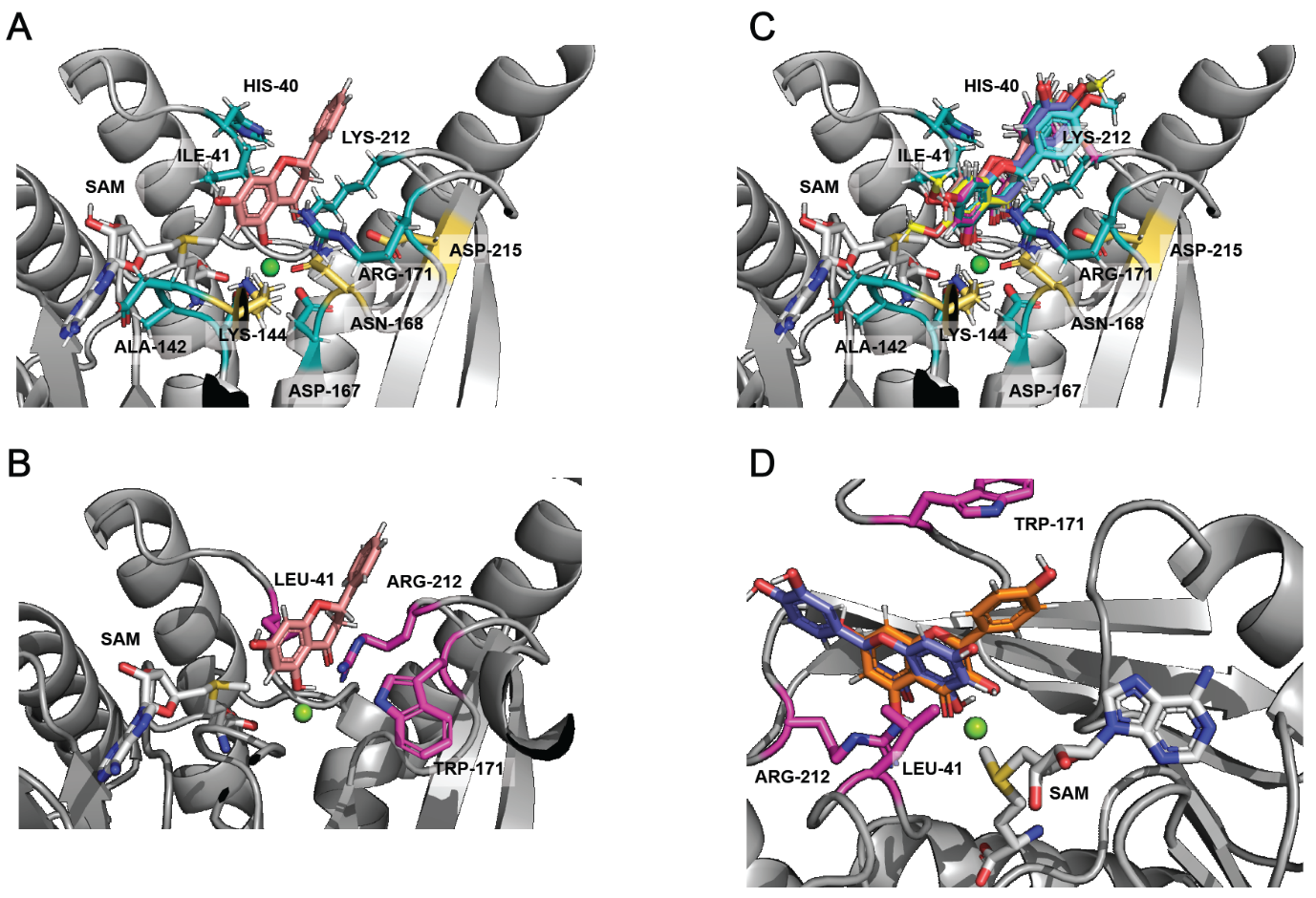


**Figure S7.** Docking models of StrAOMT (PDB ID 8C9S), where the cofactor SAH is replaced with SAM. A) binding pose of pinocembrin; B) model of the triple mutant shown with docked pinocembrin and SAM. Active site residues surrounding the docked SAM and pinocembrin are shown as stick representation with atom coloring: blue - nitrogen, red – oxygen, yellow – sulfur, grey - SAM carbon, light blue – StrAOMT carbon, pink – pinocembrin carbon).


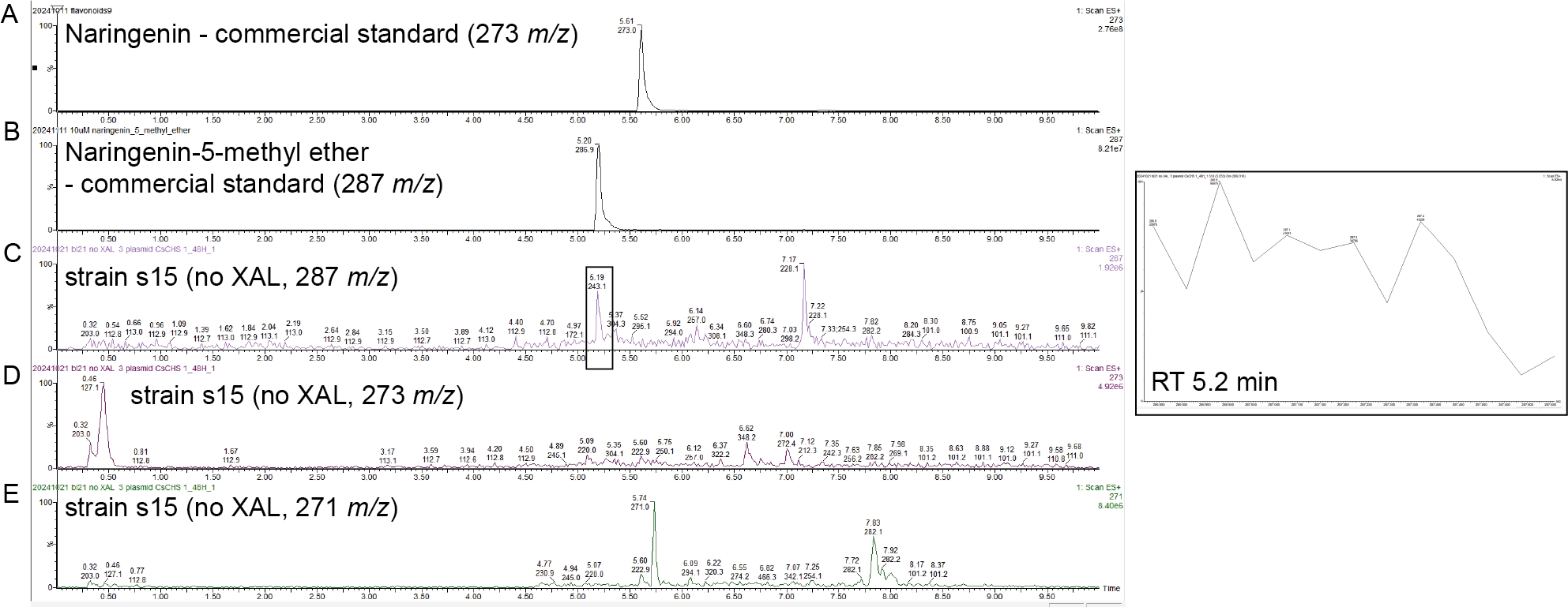


**Figure S8**. Extracted ion chromatograms of: (A) Naringenin standard compound (m/z 273 [M+H]^+^), (B) Naringenin 5-methyl ether standard compound (m/z 287 [M+H]^+^), (C) Naringenin 5-methyl ether obtained from s15 fermentation broth, (D) Naringenin obtained from s15 fermentation broth, (E) Alpintin (m/z 271 [M+H]^+^) detected in s15 fermentation broth, (F) Mass spectrum corresponding to the peak at m/z 243.1 [M+H]^+^.


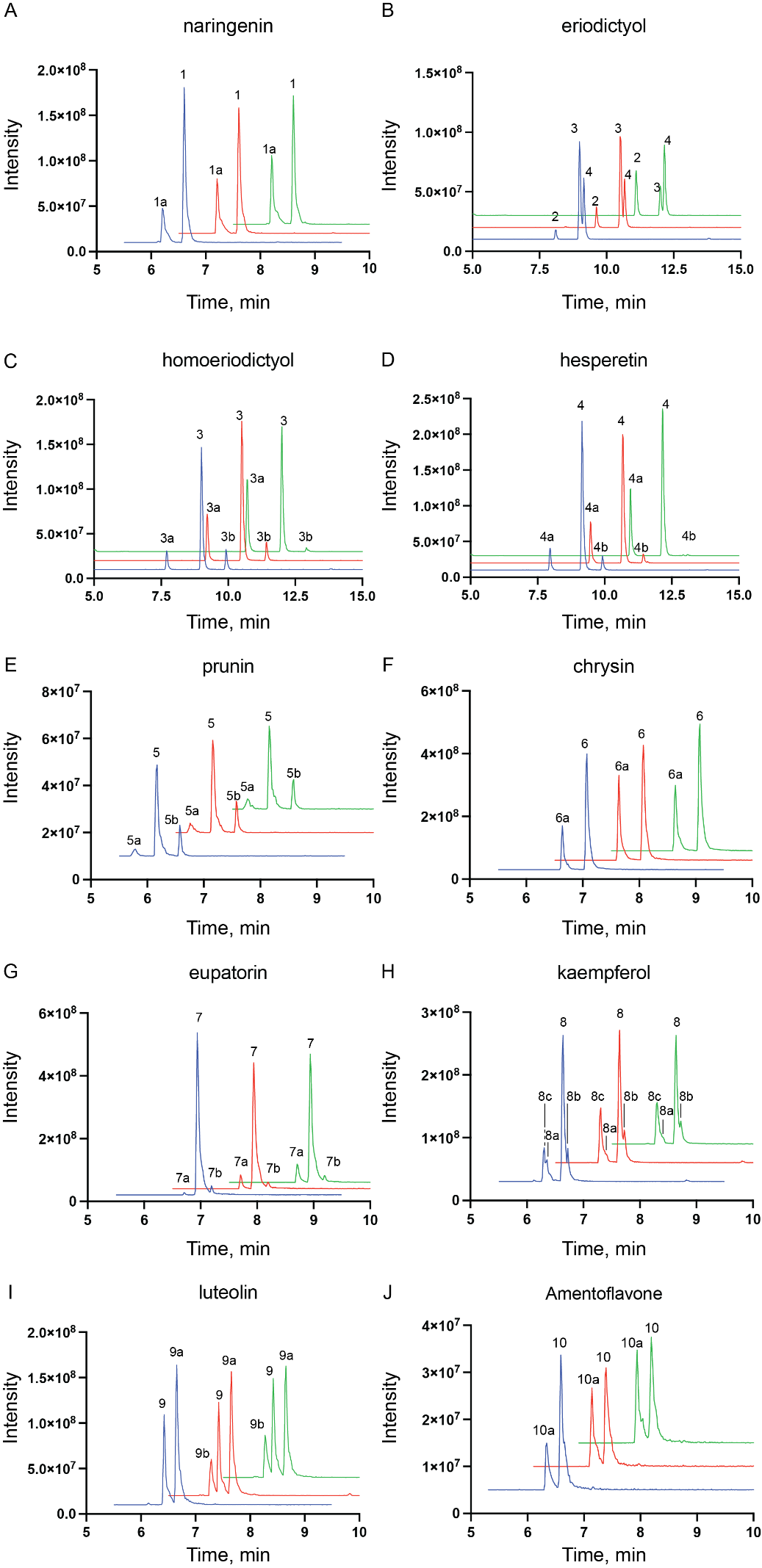


**Figure S9.** Extracted ion chromatograms of the methylated products from enzymatic reactions by StrAOMT variants analyzed by HPLC-MS. Traces of the three enzyme variants shown with an x/y offset for clarity. Blue traces: StrAOMT wildtype, red traces: StrAOMT K212R, green traces: StrAOMT I41L/R171W/K212R.


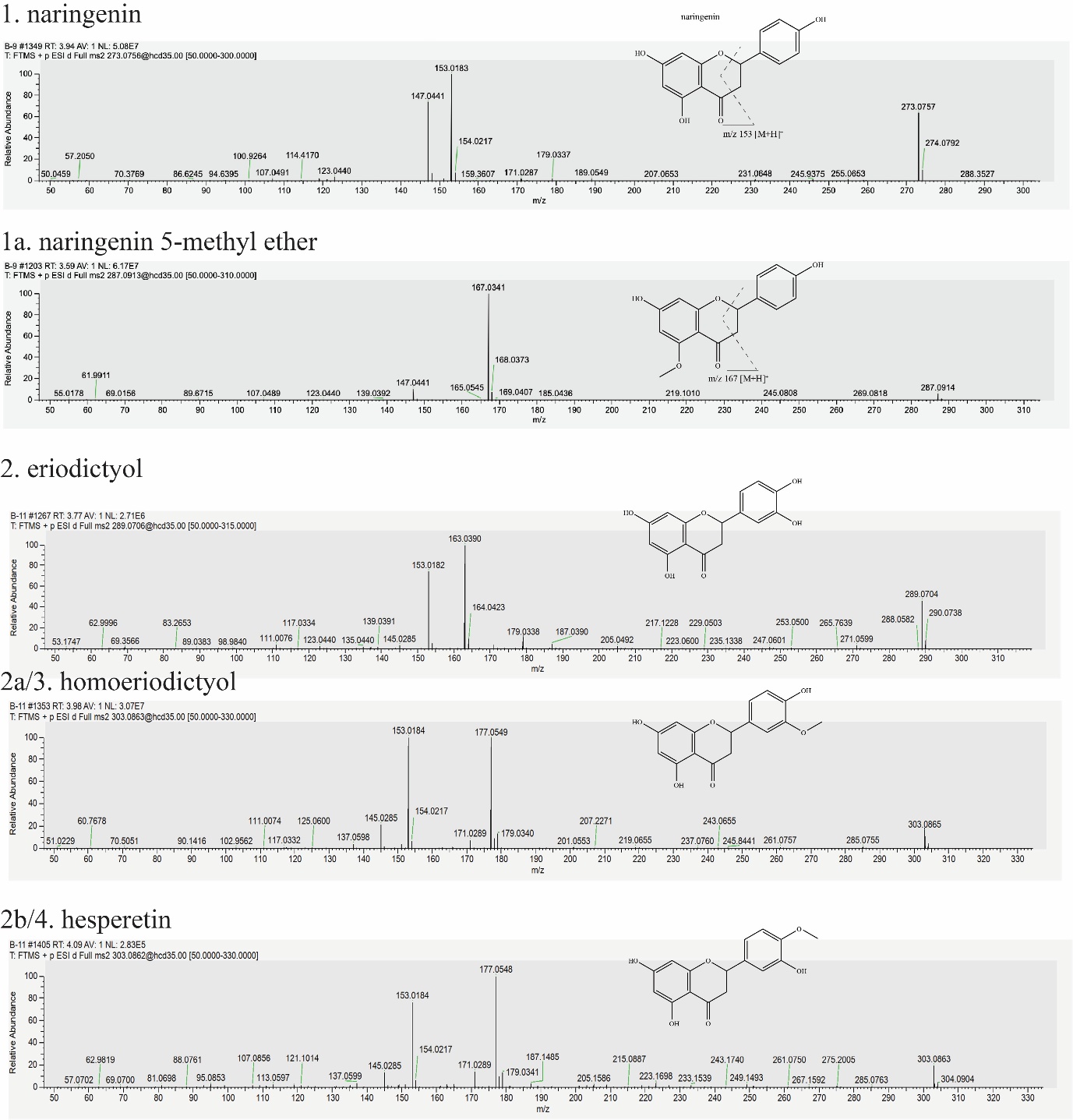

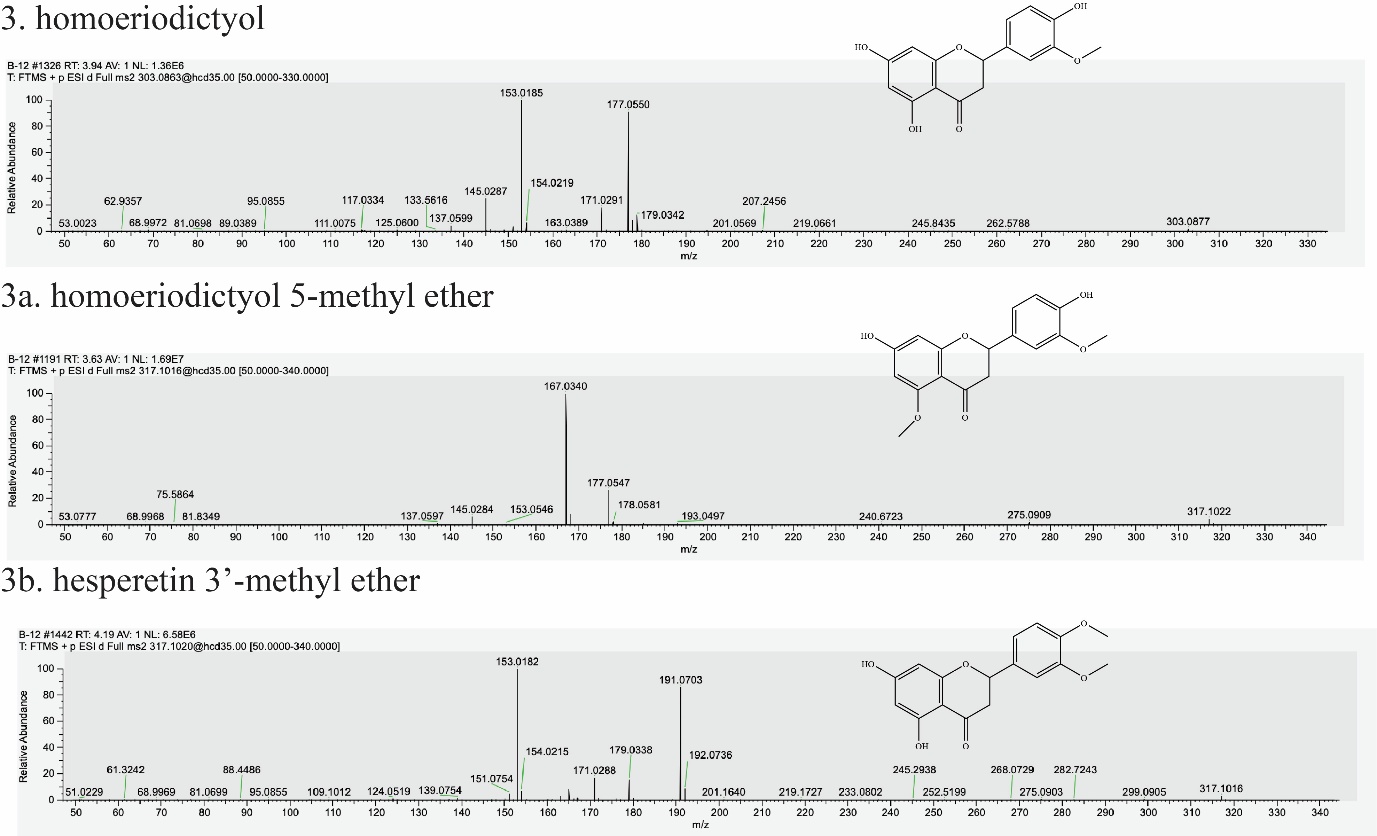

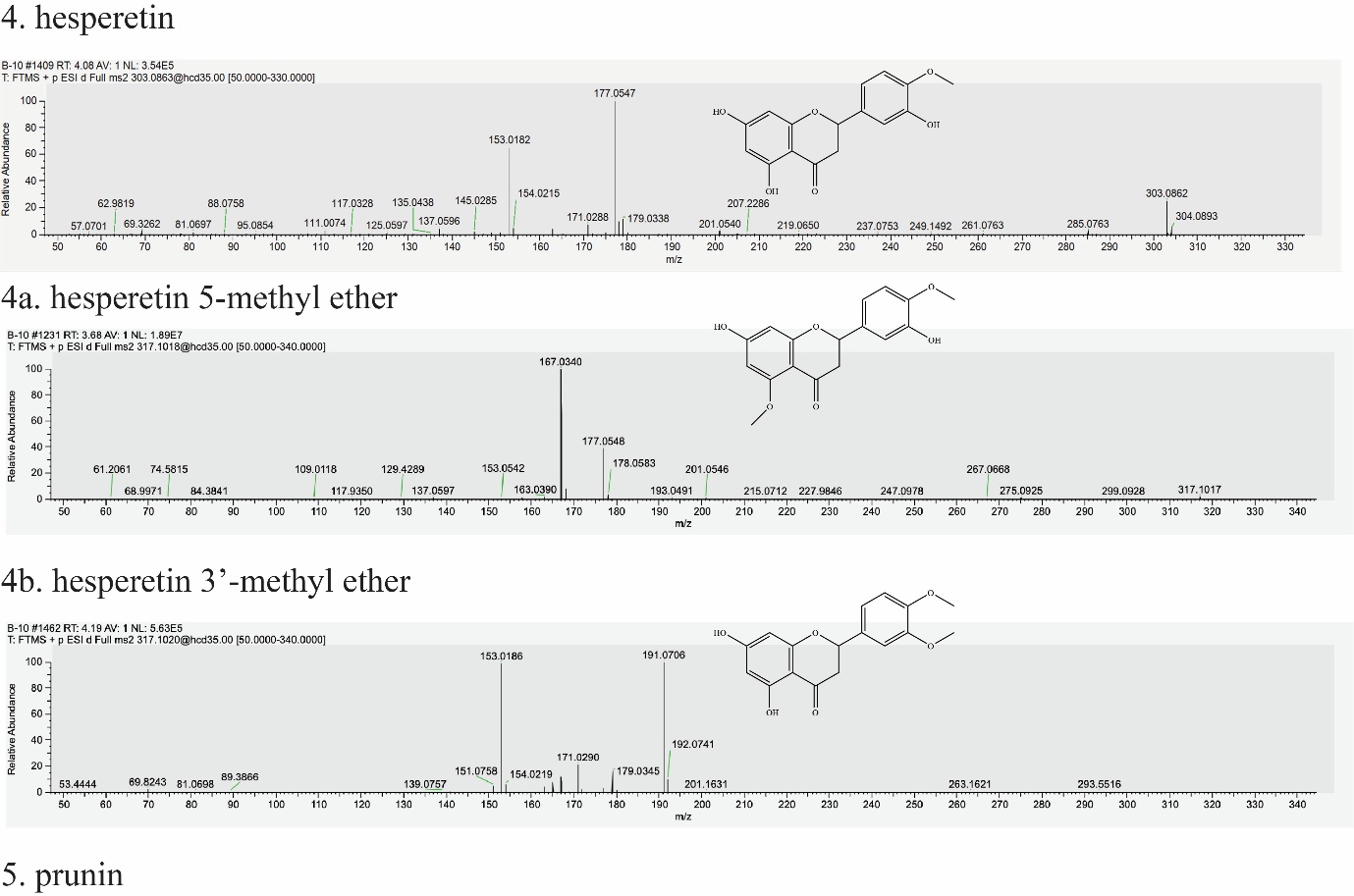

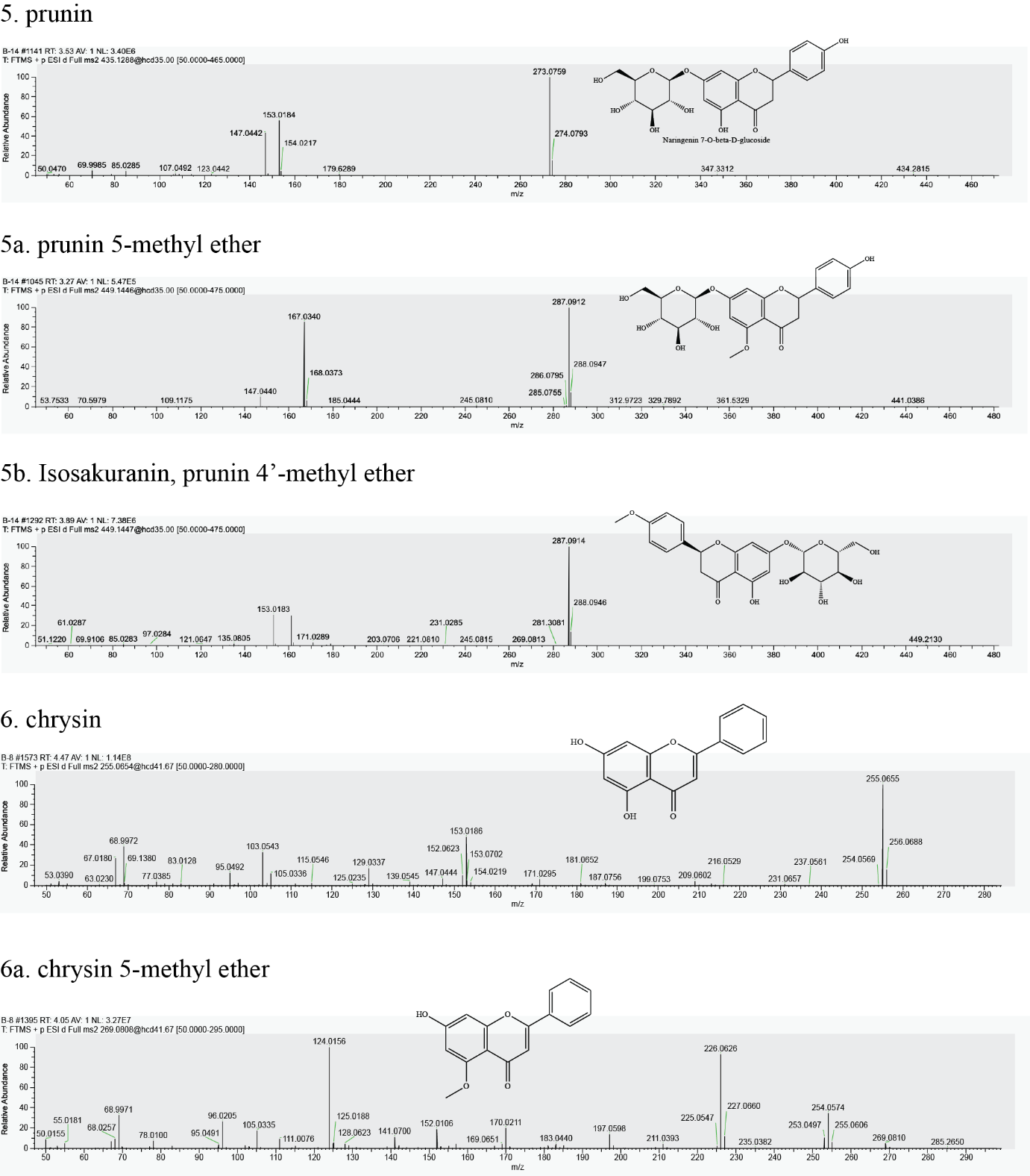


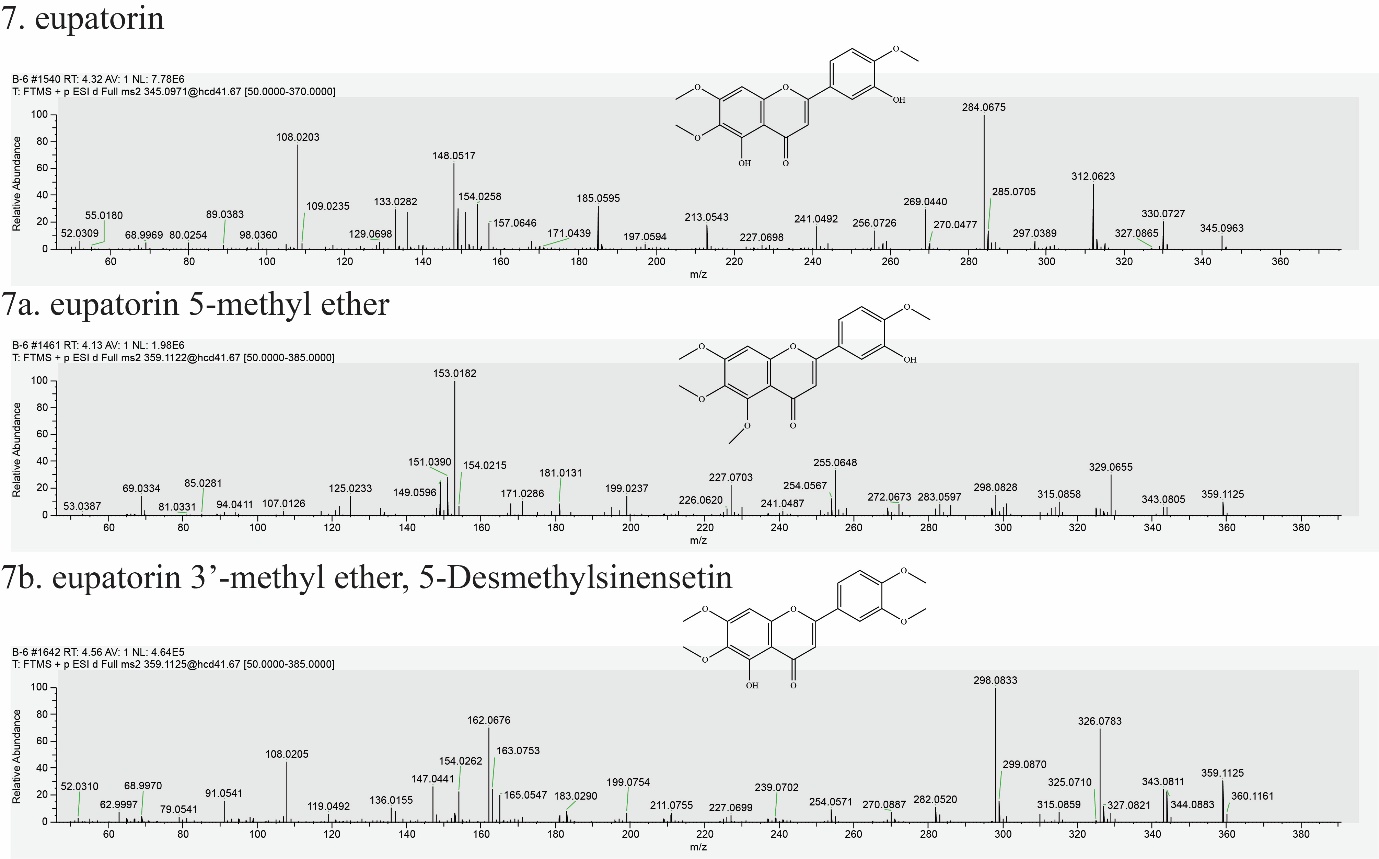

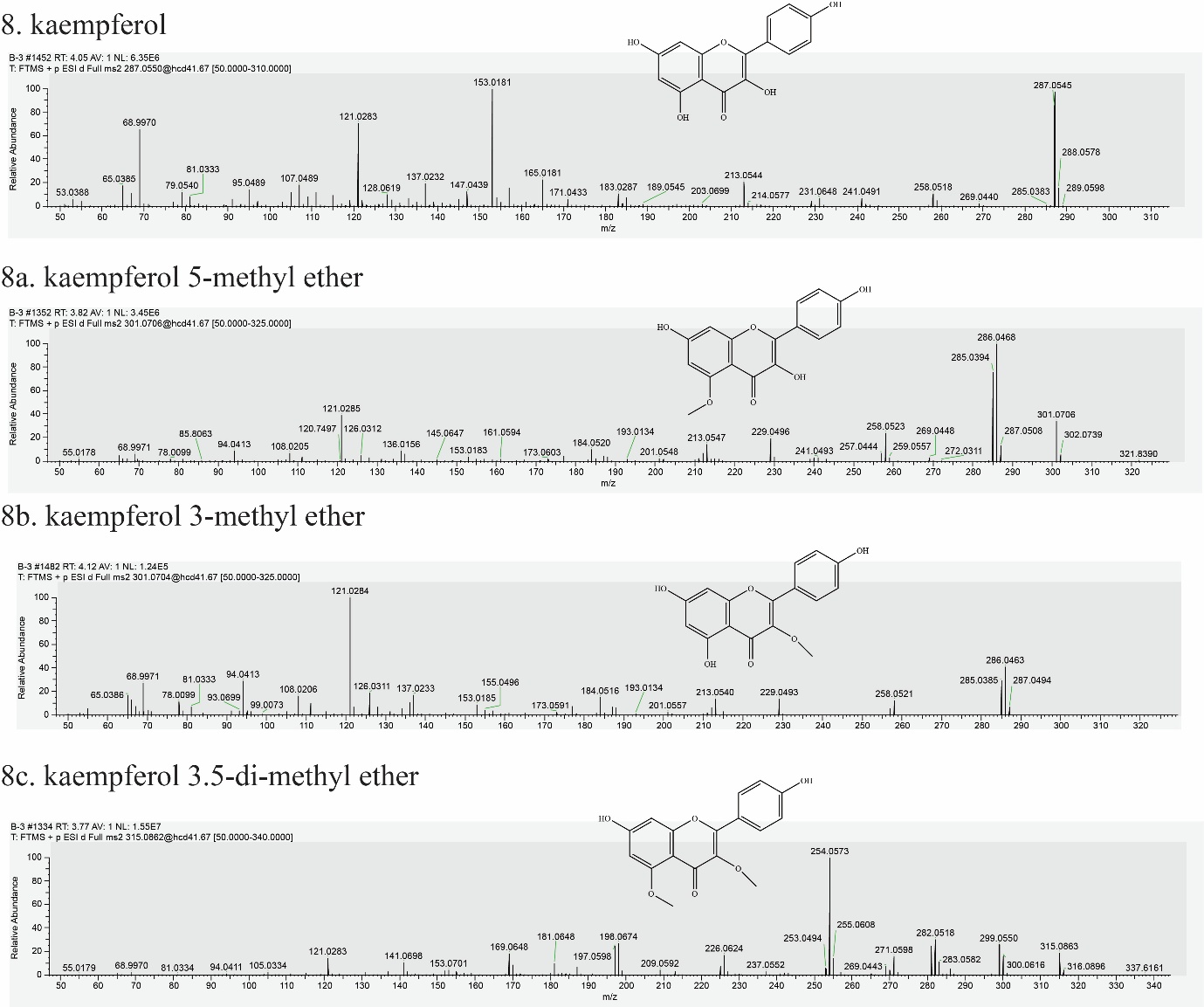

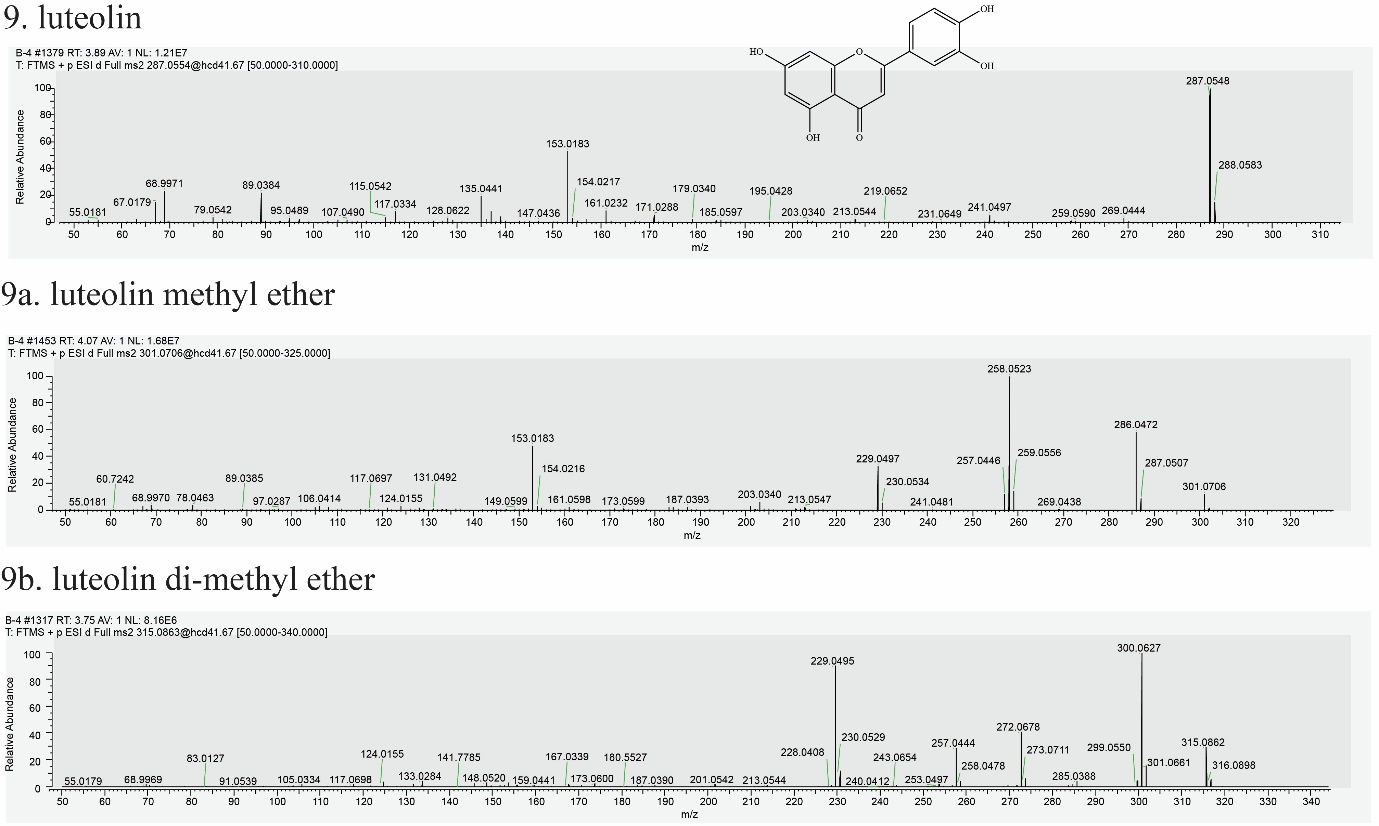

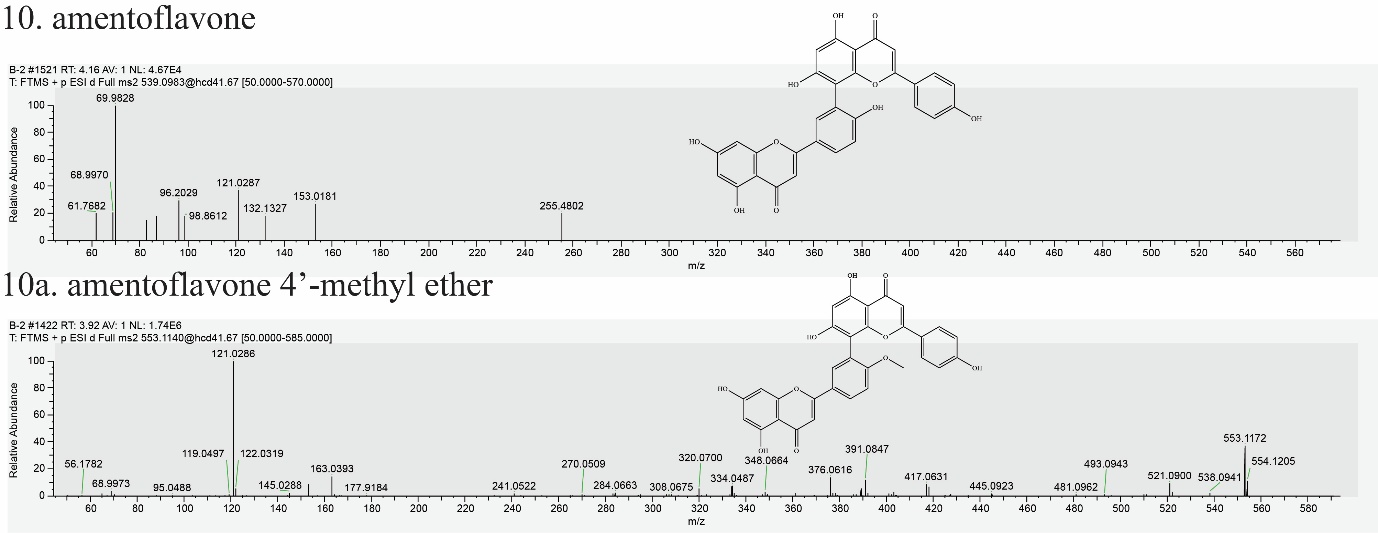

**Figure S10**. Ion mass spectra (MS2) of methylated flavonoids produced by StrAOMT variants, obtained through high-resolution tandem MS analysis.


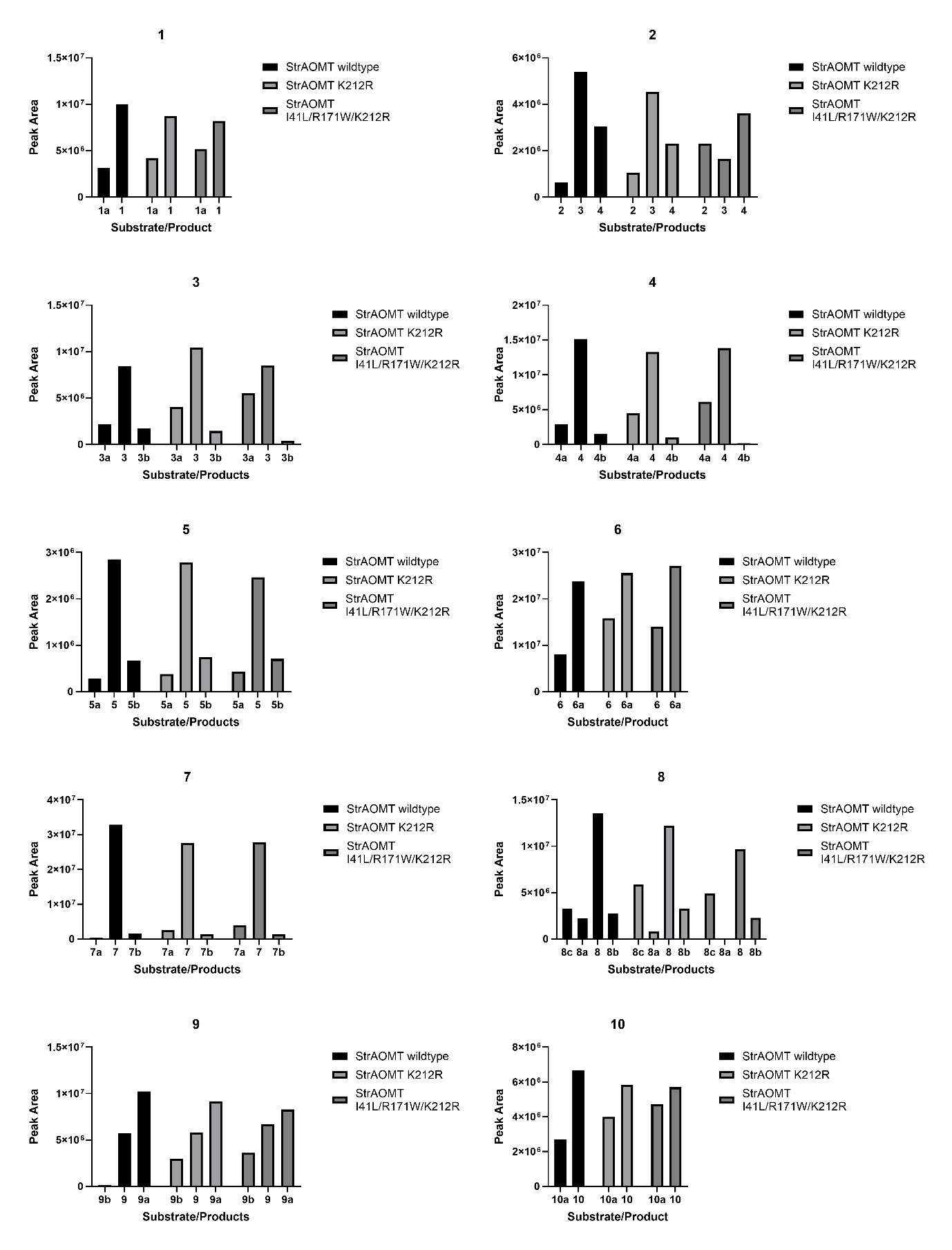


**Figure S11.** Quantification of methylated flavonoid products from enzymatic reactions by StrAOMT variants. Bar graph shows peak areas extracted from LC-MS chromatograms (Figure S8), representing apparent product formation. Black bars: StrAOMT wildtype; light gray bars: StrAOMT K212R; dark gray bars: StrAOMT I41L/R171W/K212R.


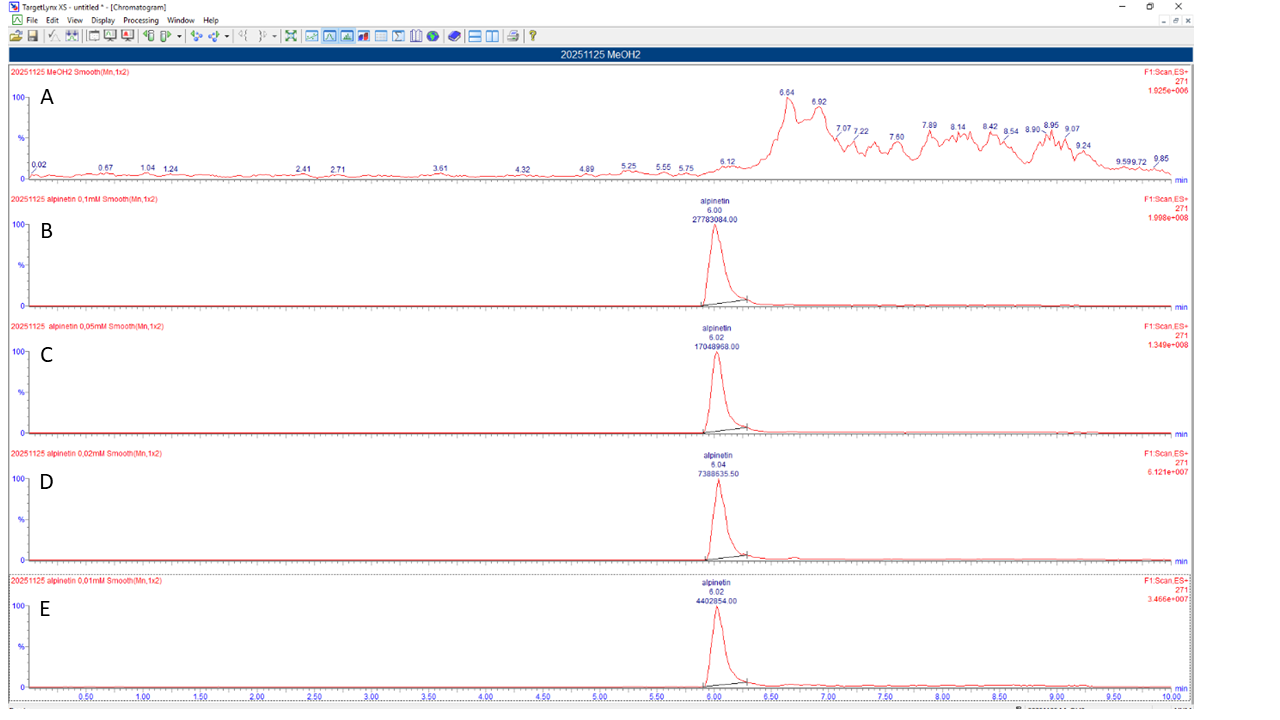


**Figure S12**. Extracted ion chromatograms and peak integrations (auto‑quantified by Waters MassLynx) of alpinetin standards at different concentrations (A–E). (A) Methanol blank. (B–E) Alpinetin standards at 0.10 mM, 0.05 mM, 0.02 mM, and 0.01 mM, respectively.
